# Methylation profiling of normal tissue adjacent to breast tumors reveals two distinct groups with divergent tumor microenvironment features

**DOI:** 10.1038/s41523-026-00944-x

**Published:** 2026-06-02

**Authors:** Hela Koka, Priscilla Ming Yi Lee, Bin Zhu, Feng Wang, Avraam Tapinos, Mustapha Abubakar, Difei Wang, Xiaoyu Wang, Xing Hua, Chad A. Highfill, Li Feng, Tongwu Zhang, Cherry Wu, Koon Ho Tsang, Yee-Kei Tsoi, W. C. Chan, Sze Hong Law, Ray Ka Wai Hung, Gary M. Tse, Kristine Jones, Aurelie Vogt, Amy Hutchinson, Belynda Hicks, Montserrat Garcia-Closas, Stephen J. Chanock, David C. Wedge, Lap Ah Tse, Xiaohong R. Yang

**Affiliations:** 1 Division of Cancer Epidemiology and Genetics, National Cancer Institute, NIH, DHHS, Bethesda, MD USA; 2 The Jockey Club School of Public Health and Primary Care, The Chinese University of Hong Kong, Hong Kong, China; 3 Manchester Cancer Research Centre, The University of Manchester, Manchester, UK; 4 Cancer Genomics Research Laboratory, Leidos Biomedical Research, Frederick National Laboratory for Cancer Research, Frederick, MD USA; 5 Department of Pathology, North District Hospital, Hong Kong, China; 6 Department of Pathology, Yan Chai Hospital, Hong Kong, China; 7 Department of Surgery, North District Hospital, Hong Kong, China; 8 Department of Anatomical and Cellular Pathology, The Chinese University of Hong Kong, Hong Kong, China

**Keywords:** Biomarkers, Cancer, Computational biology and bioinformatics, Genetics, Oncology

## Abstract

We previously identified diverse genetic evolutionary patterns in whole-genome sequencing of paired normal tissue adjacent to tumor (NAT) and tumor tissues from Hong Kong breast cancer (HKBC) patients. Here, we investigated whether DNA methylation (DNAm) contributes to NAT heterogeneity and shapes the tumor microenvironment (TME). Genome-wide DNAm profiling was performed on paired NAT and tumor tissues from 188 HKBC patients using the Infinium 850 K array. RNA-seq data were available for 76 NATs and 177 tumors. Cellular composition was inferred using MethylCIBERSORT, CIBERSORTx, and EpiDISH, and histopathologic features were assessed on 115 H&E-stained sections. Unsupervised clustering identified two distinct NAT subtypes with divergent TME characteristics. Cluster 1 (*N* = 139) showed higher epithelial and fibroblast content and enrichment of estrogen response pathways. Cluster 2 (*N* = 49) exhibited an immune-metabolic phenotype characterized by increased fat and immune cells, stromal disruption, inflammatory pathway activation, and greater macrophage infiltration. Cluster 2 patients also demonstrated significantly younger epigenetic age estimated using multiple epigenetic clocks. These DNAm-defined NAT subtypes and associated TME features were validated in 97 NAT samples from TCGA breast cancer patients. Overall, our findings identify DNAm-driven NAT heterogeneity with distinct TME landscapes, providing new insights into field cancerization and tumor evolution in breast cancer.

## Introduction

Breast cancer (BC) stands as a prominent contributor to cancer-related fatalities on a global scale. It encompasses five major subtypes: luminal A, luminal B, human epidermal receptor 2 (HER2)-enriched, basal-like, and normal-like, each characterized by distinct clinical and genomic features^1^. This intrinsic BC classification plays an important role in guiding treatment. Recent evidence underscores the importance of tumor microenvironment (TME) in cancer therapies such as immune checkpoint blockade (ICB)^2^, highlighting the importance of understanding the TME’s role in shaping tumor evolution and developing precise and effective BC treatment strategies^2^. In line with this, our recent study demonstrated that disruptions in stromal-epithelial interactions, characterized by morphological changes in tissue architecture and cell composition, were associated with increased BC risk among women with benign breast diseases and with poorer prognosis among BC patients with estrogen receptor-positive disease^3^.

Traditionally, the primary focus in assessing recurrence risk and guiding treatment decisions has revolved around scrutinizing the primary tumor’s histopathological characteristics and molecular composition. However, this approach may have overlooked the potential insights that histologically normal adjacent tissues (NATs) can provide. Recent findings suggest that exploring NATs could unveil valuable insights into the intricate characteristics of BC progression and patient prognoses^4–6^. Using data from The Cancer Genome Atlas (TCGA), Aran et al. discovered distinct gene expression and protein-protein interaction characteristics in NATs shared across various cancer types that were enriched for pro-inflammatory signals^6^. Similarly, another study using TCGA data revealed that NATs provided more informative insights on patient survival than tumor samples alone and that genes associated with survival outcomes were enriched with immune-related pathways^4^. Moreover, previous research has demonstrated that NATs contain genetic, epigenetic, and transcriptomic alterations^6–8^ that are likely early indicators of breast carcinogenesis, aligning with the field cancerization hypothesis. This concept describes morphologically “normal” tissue regions that contain precancerous cells with underlying molecular alterations^9^. Therefore, analyzing genetic and epigenetic changes in NATs can reveal information about an individual’s cumulative exposure to environmental factors that may contribute to cancer development and progression ^7^.

We previously examined the genomic features of NATs using whole-genome sequencing (WGS), methylation profiling, and RNA sequencing (RNA-seq) from 43 breast cancer patients in Hong Kong^10^. Our findings revealed that NATs frequently contained single-nucleotide variants (SNVs) in key driver genes, many of which were also present in paired tumor samples, aligning with the concept of field cancerization. Notably, when generating phylogenetic trees to explore the evolutionary history of NAT and tumor samples, we identified distinct patterns of mutations in paired NAT and tumor samples. For example, NAT samples in the multiple-tree group, characterized by separate tree structures in tumor and NAT samples, demonstrated a less inflammatory and more suppressive TME, compared to the shared-tree group, where tumor and NAT samples shared at least part of a single tree structure. While this research provided valuable insights, it was constrained by a small sample size, underscoring the need for a more comprehensive investigation of normal tissue to better understand its implications for cancer development and patient outcomes. In the current analysis, we aimed to further characterize the molecular heterogeneity of NATs by integrating methylation and gene expression profiling data from an expanded sample set. This approach seeks to deepen our understanding of field cancerization and tumor evolution.

## Results

### Study population

This study involved 188 treatment-naïve Chinese women in Hong Kong diagnosed with invasive breast cancer, from whom fresh-frozen histologically normal adjacent tissues (NATs) and paired breast tumor tissue samples were collected. Most NAT samples were collected from a distance greater than 2 cm from the tumor and were evaluated by two independent pathologists, confirming their histologically normal nature. RNA-seq data were available for 76 NAT and 177 tumor samples, respectively. The mean age at diagnosis was 59.9 years (SD = 11.1, range = [31.0, 84.0]), and the mean body mass index (BMI) was 25.0 kg/m² (SD = 4.1, range = [14.5, 39.0]). All NAT samples except one (basal-like) were classified as normal-like, while the tumor subtype distribution was as follows: 37% luminal A (*N* = 66), 28% luminal B (*N* = 50), 15% HER2-enriched (*N* = 27), 12% basal-like (*N* = 21), and 7% normal-like (*N* = 13), according to PAM50^11^. Additional patient characteristics are presented in Table 1.

**Table 1:** Patient characteristics in 188 Hong Kong breast cancer patients

Characteristic	Overall (*N* = 188) *N* (%)	Cluster 1 (*N* = 139) *N* (%)	Cluster 2 (*N* = 49) *N* (%)	*P*-value^a^
Age, year				
Mean (SD)	59.9 (11.1)	59.7 (11.5)	60.5 (9.8)	0.470
<50	35 (18.6)	30 (21.6)	5 (10.2)	0.209
50–60	53 (28.2)	38 (27.3)	15 (30.6)	
≥60	100 (53.2)	71 (51.1)	29 (59.2)	
Missing				
BMI^b^, kg/m^2^				
Mean (SD)	25.0 (4.1)	25.1 (4.3)	24.8 (3.5)	0.940
<25	93 (56.4)	69 (55.7)	24 (58.5)	0.791
25–30	50 (30.3)	37 (29.8)	13 (31.7)	
≥30	22 (13.3)	18 (14.5)	4 (9.8)	
Missing	23	15	8	
Age at menarche, year				
Mean (SD)	13.7 (2.0)	13.6 (2.0)	13.9 (2.0)	0.450
<14	89 (53.6)	66 (54.1)	23 (52.3)	0.862
≥14	77 (46.4)	56 (45.9)	21 (47.7)	
Missing	22	17	5	
Parity				
Nulliparous	17 (9.4)	10 (7.5)	7 (14.6)	0.158
Parous	164 (90.6)	123 (92.5)	41 (85.4)	
Missing	7	6	1	
Menopausal status				
Pre	39 (21.3)	33 (24.4)	6 (12.5)	0.102
Post	144 (78.7)	102 (75.6)	42 (87.5)	
Missing	5	4	1	
Age of menopause, year^c^				
Mean (SD)	49.6 (4.9)	49.8 (5.1)	49.1 (4.5)	0.190
≤50	72 (58.5)	48 (55.8)	24 (64.9)	0.426
>50	51 (41.5)	38 (44.2)	13 (35.1)	
Missing	21	16	5	
PAM50^d^				
Luminal A	66 (37.3)	48 (37.2)	18 (37.5)	0.993
Luminal B	50 (28.2)	36 (27.9)	14 (29.2)	
HER2-enriched	27 (15.3)	20 (15.5)	7 (14.6)	
Basal-like	21 (11.9)	16 (12.4)	5 (10.4)	
Normal-like	13 (7.3)	9 (7.0)	4 (8.3)	
Missing	11	10	1	
Clinical grade				
1	22 (18.8)	17 (20.2)	5 (15.2)	0.799
2	52 (44.4)	36 (42.9)	16 (48.5)	
3	43 (36.8)	31 (36.9)	12 (36.4)	
Missing	71	55	16	

^a^*P*-value obtained from Chi-squared, Fisher’s Exact or Wilcoxon test.

^b^*BMI* body mass index.

^c^Among menopausal women.

^d^AIMS was used to infer tumor subtype using RNA-seq data.

### Methylation-based subtypes and cellular composition

**Fig. 1:**
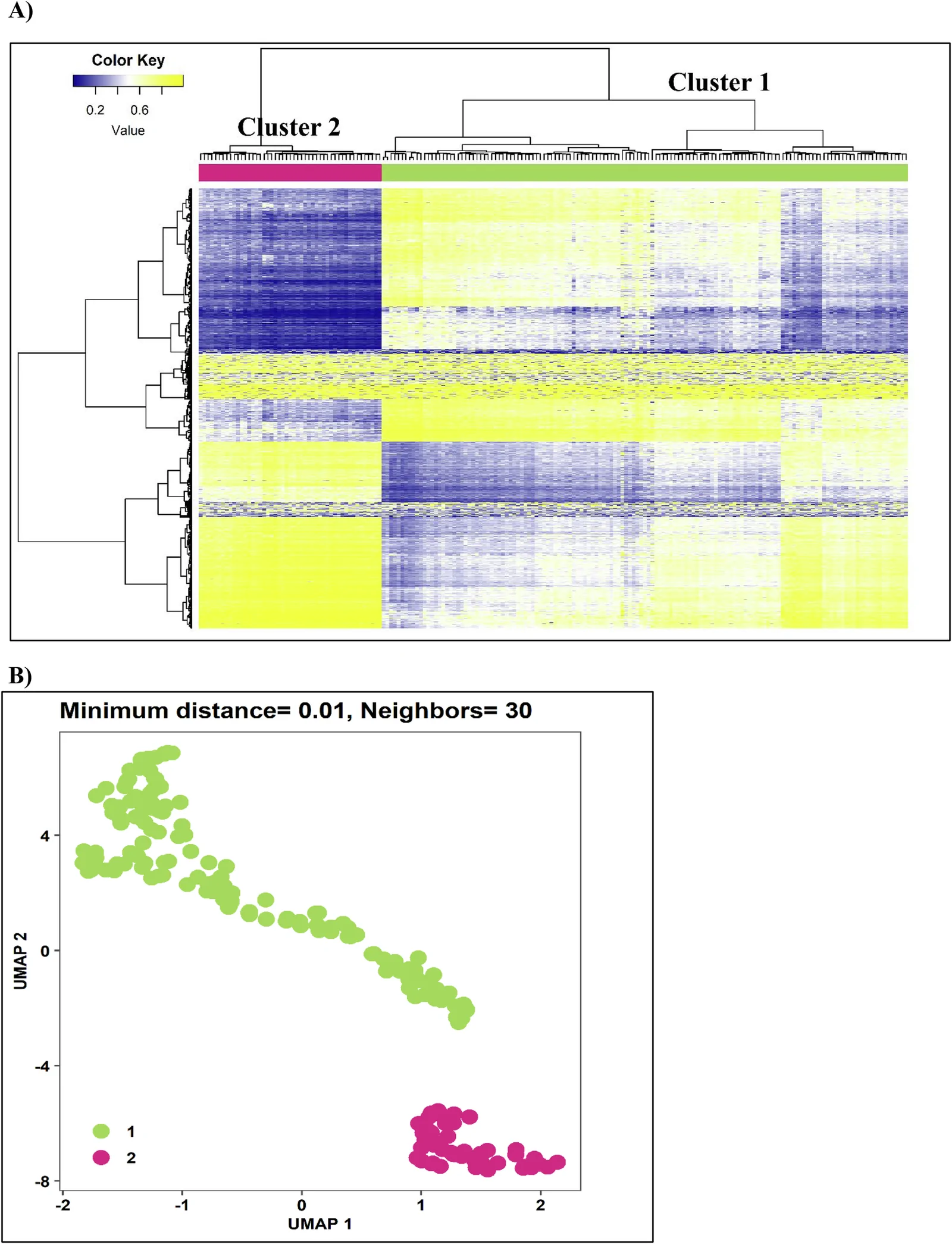
Characterization of methylation profiles from adjacent normal tissue samples collected from 188 breast cancer patients. **A** Unsupervised clustering analysis based on the top 10,000 most variable methylation probes; samples are represented as columns and methylation probes as rows, with beta values displayed using a color gradient from navy (*β* = 0) to yellow (*β* = 1) to indicate increasing methylation; **B** UMAP plot of clustering with n_neighbors = 30 and min_dist = 0.01.

To assess the molecular heterogeneity of NAT samples, we conducted consensus clustering using the hierarchical clustering method on methylation profiling data from 188 NAT samples, which indicated *k* = 2 as the optimal cluster solution (see “Methods” and Supplementary Fig. 2). We then performed unsupervised clustering analysis and identified two distinct clusters (Fig. 1A) and applied UMAP to visualize the underlying patterns in two-dimensional space (Fig. 1B).

**Fig. 2:**
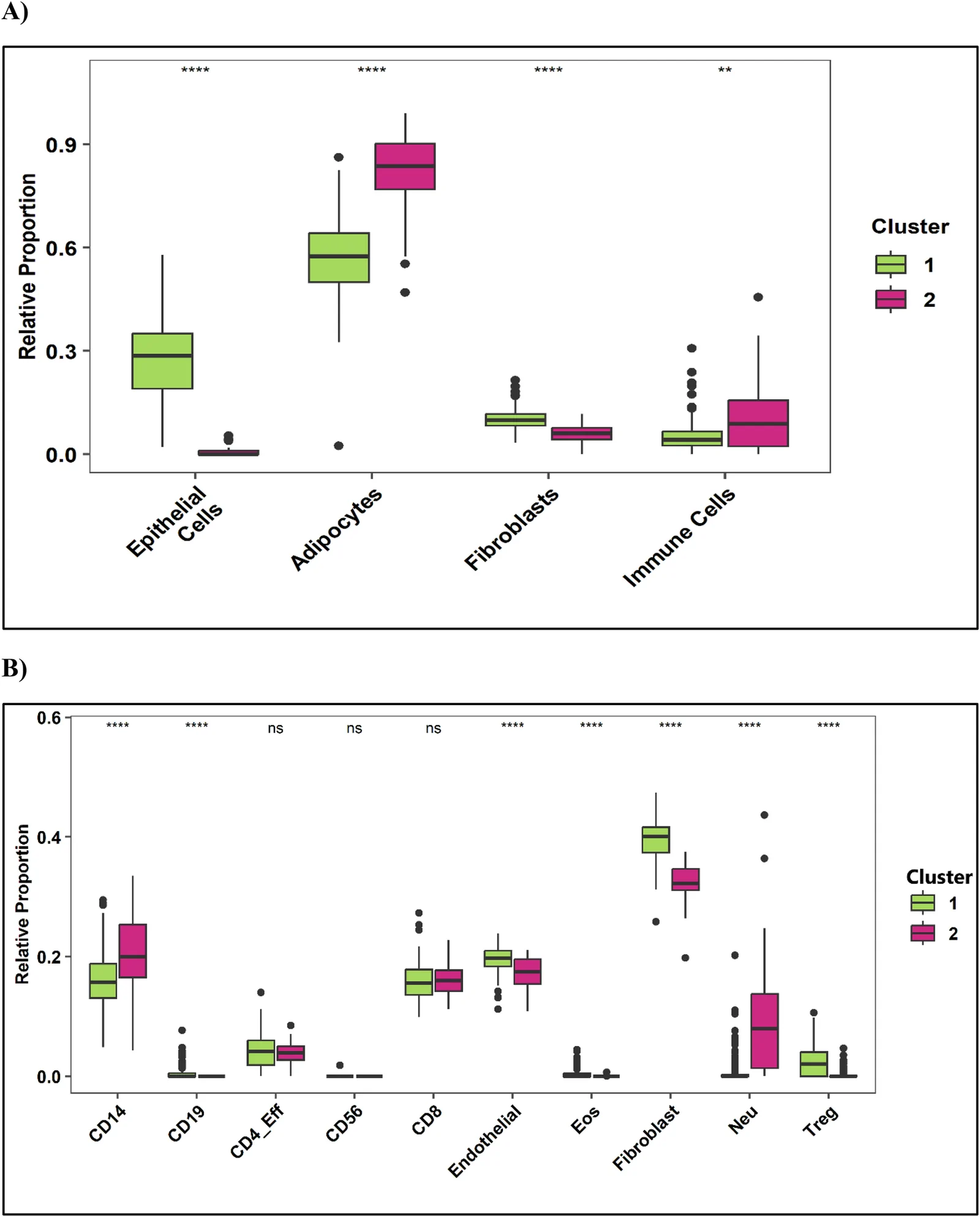
Comparison of cell composition in adjacent normal tissue samples between the two methylation clusters. **A** Proportions of cell types inferred by EpiDISH; **B** Proportions of cell types inferred by MethylCIBERSORT. Significance levels are denoted as follows: ***P-value < 0.001, **P-value < 0.01, *P-value < 0.05.

The first cluster (Cluster 1, containing most samples (*N* = 139)) was characterized by significantly higher epithelial and fibroblast content as estimated by EpiDISH (Fig. 2A). In contrast, Cluster 2 (*N* = 49) demonstrated a different profile, with samples showing lower content of epithelium but higher tissue fat and immune cell levels (Fig. 2A). When comparing the TME cell composition, as estimated by the deconvolution algorithm MethylCIBERSORT, we found that Cluster 2 samples showed higher proportions of CD14, neutrophils, and lower fractions of endothelial cells, Tregs, and fibroblasts than Cluster 1 samples (Fig. 2B). The distance between NAT and paired tumor samples did not vary significantly between Cluster 1 ( < 2 cm: 14%; 2-4 cm: 28%; ≥4 cm: 58%) and Cluster 2 ( < 2 cm: 17%; 2–4 cm: 26%; ≥4 cm: 57%) (Supplementary Fig. 3). We also observed a consistent classification (with 100% concordance) using gene expression data in NAT samples with RNA-seq data (see Supplementary Fig. 4).

### Clinical, patient, and molecular factors associated with methylation-based subtypes

**Fig. 3:**
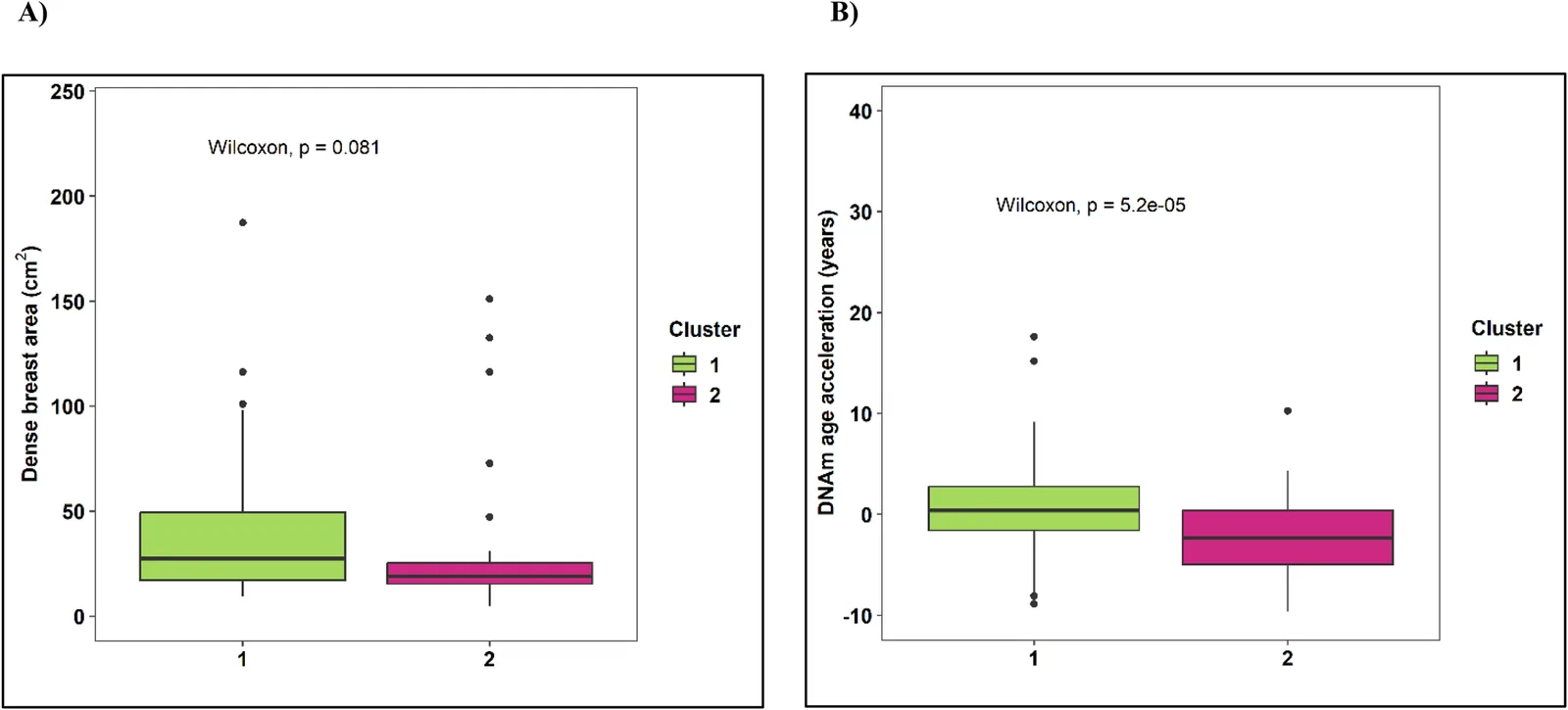
Comparison of patient characteristics between the two methylation clusters. **A** Dense breast area (cm^2^) estimated by LIBRA software; **B** DNA methylation (DNAm) age acceleration (years) estimated in adjacent normal tissue samples, using Horvath epigenetic clock. DNAm age acceleration was estimated to be the residual from regressing DNA methylation age on chronological age.

Age, tumor PAM50 subtype, and major breast cancer risk factors such as parity, age at menarche, BMI, menopausal status, and age at menopause did not vary significantly between patients in the two clusters (Table 1), although patients in Cluster 2 tended to have lower average dense breast areas (34.85 cm^2^) compared to those in Cluster 1 (41.43 cm^2^, Wilcoxon test; *P* = 0.081, Fig. 3A) in a subset of patients with continuous mammographic density data (*N* = 70). Interestingly, Cluster 2 patients had significantly younger epigenetic age, estimated based on the Horvath clock, compared to Cluster 1 patients (Fig. 3B), which may better capture tissue aging than chronological age. This pattern of reduced epigenetic age acceleration in Cluster 2 was consistent across the Hannum and Levine clocks (*P* = 0.00029 and *P* < 2e-16, respectively; Supplementary Fig. 5). Consistently, NAT samples in Cluster 2 had significantly lower expression of *CDKN2A* (Wilcoxon test; *P* = 1.8e-08) compared to those in Cluster 1, a known aging and senescence marker (Supplementary Fig. 6).

### Morphological features of methylation-based subtypes

**Fig. 4:**
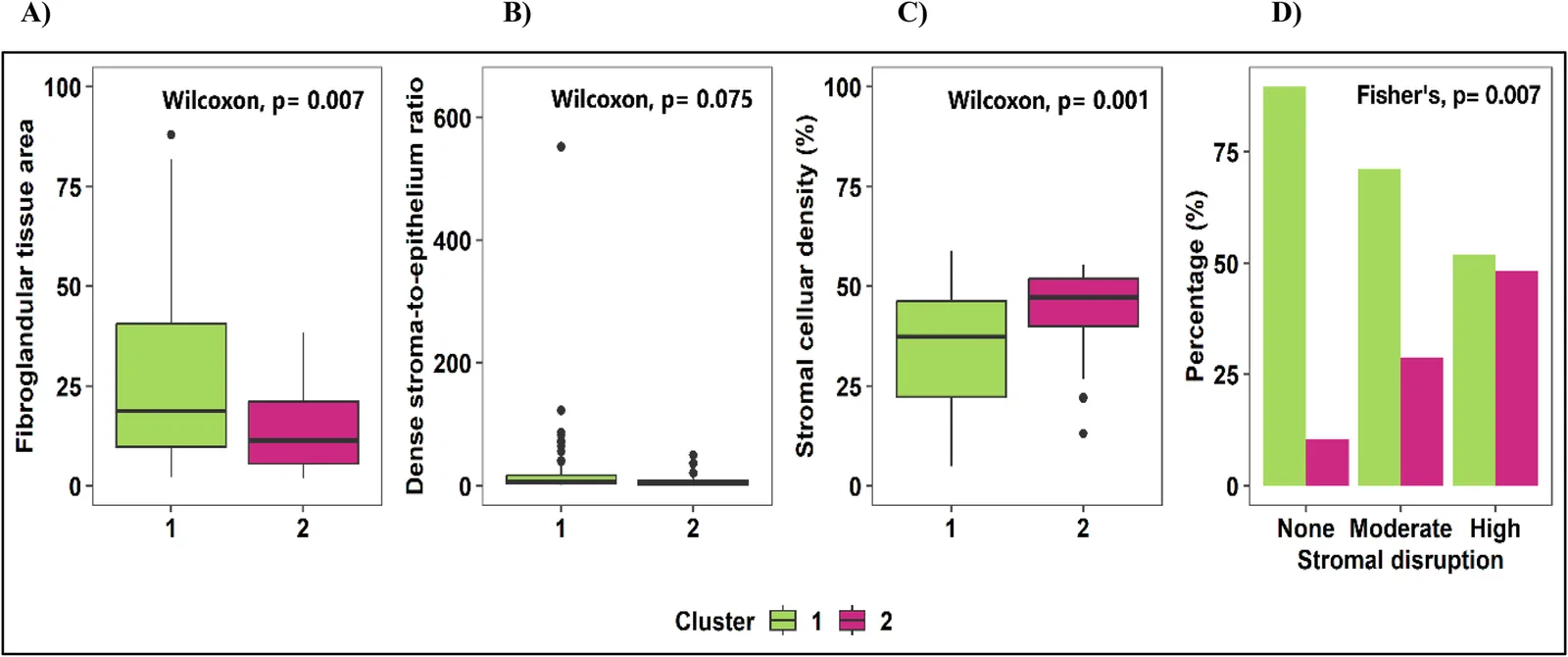
Comparison of tissue morphology phenotypes between the two methylation clusters on H&E-based images. **A** Fibroglandular tissue area, **B** dense stroma-to-epithelium ratio, **C** stromal cellular density, and **D** stromal disruption distributions.

To assess whether NAT samples in the two clusters differed in morphology, we examined the relationships between NAT clusters and machine learning-based tissue features (*N* = 115). Supervised machine learning algorithms were applied to H&E-stained sections to quantify fibroglandular tissue content, dense stroma-to-epithelium ratio, and stromal cellular density; these latter two measures were used to classify stromal disruption as none/minimal, moderate, or substantial, as previously described^3^. Compared to Cluster 1, NAT samples in Cluster 2 demonstrated lower fibroglandular tissue content (Wilcoxon test; *P* = 0.007), higher stromal cellular density (*P* = 0.001), a suggestive reduction in dense stroma-to-epithelium ratio (Wilcoxon test; *P* = 0.075), and a higher degree of stromal disruption (Fisher’s Exact test; *P* = 0.007) (Fig. 4). Representative images for selected samples from each cluster are shown in Supplementary Fig. 7.

### Immune cell composition and inflammatory markers in methylation-based subtypes

Consistent with MethylCIBERSORT estimates, Cluster 2 NAT samples had higher proportions of the CD14+ cell population compared to Cluster 1 NAT samples (Fig. 2B). Correspondingly, these samples showed increased expression levels of CD14 downstream inflammatory markers, including *IL-6*, *IL-8*, and *CCL3* (Supplementary Fig. 8). Furthermore, among NAT samples with available gene expression data, we observed higher fractions of monocytes, M2 macrophages, and neutrophils in Cluster 2, along with much lower content of CD8 + T cells based on CIBERSORTx (Supplementary Fig. 9). Additionally, Cluster 2 samples showed upregulation of macrophage markers such as *CD68* and *CD163* (Supplementary Fig. 10) than Cluster 1 samples. These results suggest a more chronic inflammatory environment within Cluster 2 compared to Cluster 1 NAT samples.

Among the 41 patients with whole genome sequencing (WGS) data from our previous phylogenetic tree analysis ^10^, 31 samples were classified as Cluster 1, while 10 were classified as Cluster 2. Cluster 2 patients were more likely to exhibit shared mutations between paired tumor and NAT samples (shared-tree group, 40.0% vs. 32.3% in Cluster 1), while Cluster 1 patients more frequently showed distinct evolutionary trajectories in matched NAT and tumor samples (multiple-tree group, 58.1% vs. 20.0% in Cluster 2; Supplementary Fig. 11, Fisher’s Exact test; *P* = 0.034) compared to Cluster 2. In our previous analysis, NAT samples in the sharedtree group were associated with a higher number of mutations and a more inflammatory TME, which are consistent with the findings in the current study. Together, these results suggest that molecular heterogeneity in NATs, including both genetic and epigenetic alterations, is linked to tumor evolution processes and inter- and intra-tumoral heterogeneity.

### Pathway enrichment analysis

**Fig. 5:**
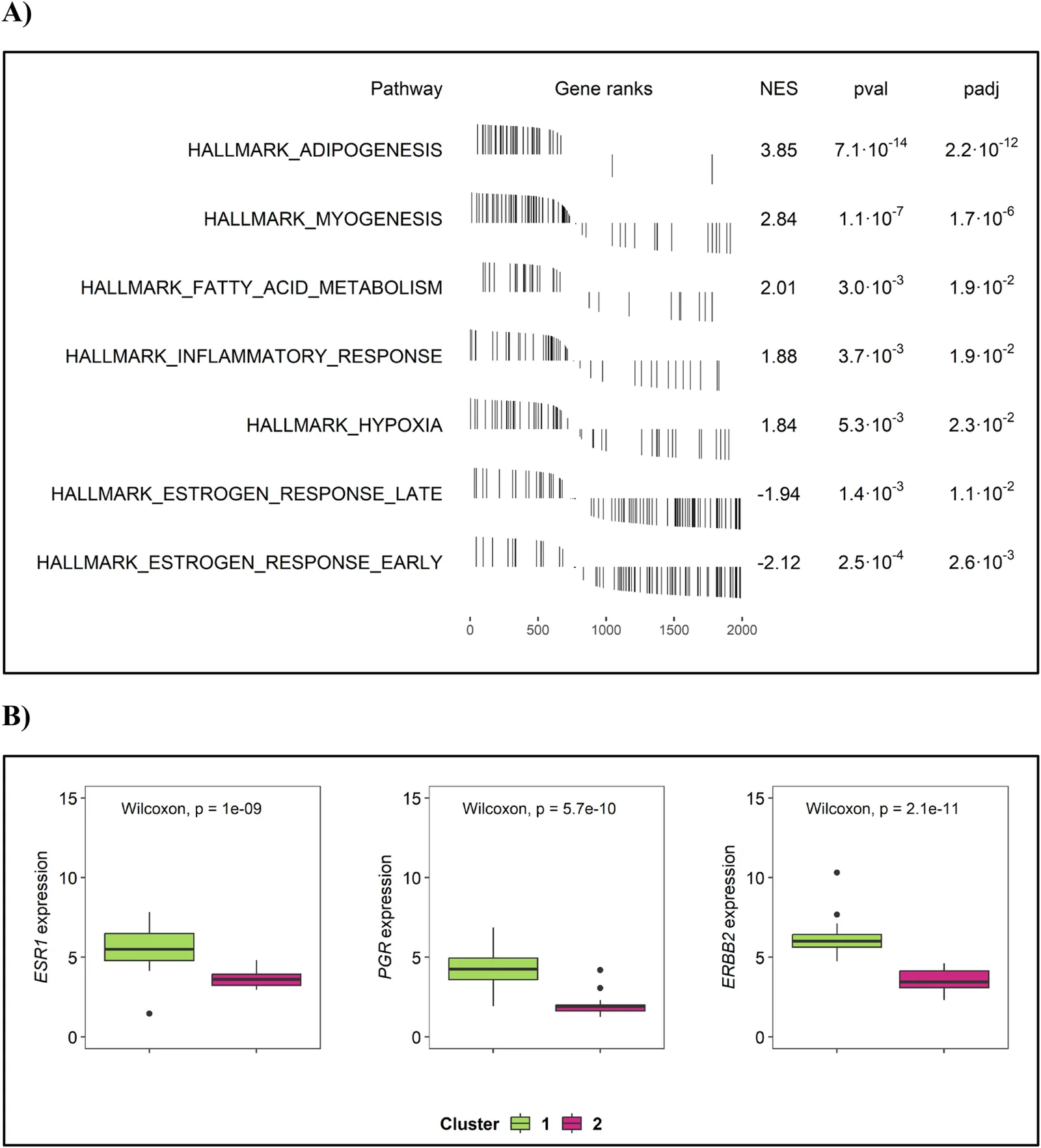
Differentially expressed genes and enriched pathways between the two methylation clusters. **A** Fast Gene Set Enrichment (FGSEA) analysis of differentially expressed genes using Hallmark gene sets from The Molecular Signatures Database (MSigDB) comparing Cluster 2 withs Cluster 1 samples; **B** Gene expression levels of *ESR1*, *PGR*, and *ERBB2* in adjacent normal tissue samples by cluster.

We conducted fast gene set enrichment analysis (FGSEA) on differentially expressed genes using 50 Hallmark gene sets^12^ comparing Cluster 2 with Cluster 1, and identified seven significantly enriched pathways (FDR < 0.05, Fig. 5A). Consistent with the higher epithelial content observed in Cluster 1 and the more inflammatory TME in Cluster 2, estrogen response pathways were upregulated in Cluster 1 NAT samples, while the inflammatory response pathway was enriched in Cluster 2 NAT samples (Fig. 5A). In addition, Cluster 2 NAT samples showed upregulation of pathways associated with adipogenesis, myogenesis, fatty acid metabolism, and hypoxia (Fig. 5A, Supplementary Table 1). Consistent with the pathway analysis data, we found elevated *ESR1* and *PGR* expression levels in NAT samples in Cluster 1 NAT samples (Fig. 5B).

### Promoter methylation of breast cancer-related genes across the methylation-based subtypes

To further investigate epigenetic mechanisms underlying the transcriptional differences between NAT clusters, we examined promoter methylation (TSS probes in CpG islands) of selected breast cancer-relevant genes (*RASSF1*, *HOXA5, and BRCA1;* Supplementary Fig. 12), whose promoter methylation has been implicated in breast cancer development and progression. Promoter methylation of the tumor suppressor *RASSF1* was elevated in Cluster 1 NAT samples and further increased in tumor tissues, consistent with its association with epithelial-rich and tumor-proximal states. In contrast, Cluster 2 NAT samples showed increased promoter methylation of *HOXA5*, a gene involved in tissue differentiation and stromal organization, aligning with the inflammatory and adipose-enriched microenvironment characteristic of this cluster. Cluster 2 NAT samples also exhibited higher promoter methylation of *BRCA1*, a key homologous recombination repair gene whose alterations of which have been linked to genomic instability. Notably, this finding is concordant with the higher mutational burden and younger epigenetic age observed in Cluster 2 NATs. Promoter methylation of another tumor suppressor, *CDH1*, encoding the cell adhesion molecule E-cadherin, did not differ between the two clusters.

### Exploratory three-cluster model

To assess whether additional substructure existed beyond the primary two-cluster framework (Fig. 1A), we explored a three-cluster model. The three-cluster solution subdivided the original Cluster 1 (*N* = 139) into sub-Cluster 1A (*N* = 104) and sub-Cluster 1B (*N* = 35) (Supplementary Fig. 13). In this model, sub-Cluster 1B displayed intermediate features between sub-Cluster 1A and Cluster 2, including relatively greater stromal cellular density, altered immune and fibroblast composition, and graded shifts in *ESR1*, *PGR*, and *ERBB2* expression compared with sub-Cluster 1A, consistent with a transitional position along the immune-stromal axis. DNA methylation age-acceleration measures also differed across the three clusters, though these differences followed the same overall continuum observed in the two-cluster model. Importantly, the three-cluster solution resolved additional heterogeneity within the epithelial-enriched group but did not introduce a new biological axis. The principal distinction between epithelial-enriched and immune-metabolic phenotypes remained intact, supporting the use of the two-cluster framework for primary downstream analyses while acknowledging additional granularity within the epithelial spectrum.

### Validation of methylation-based subtypes in TCGA

**Fig. 6:**
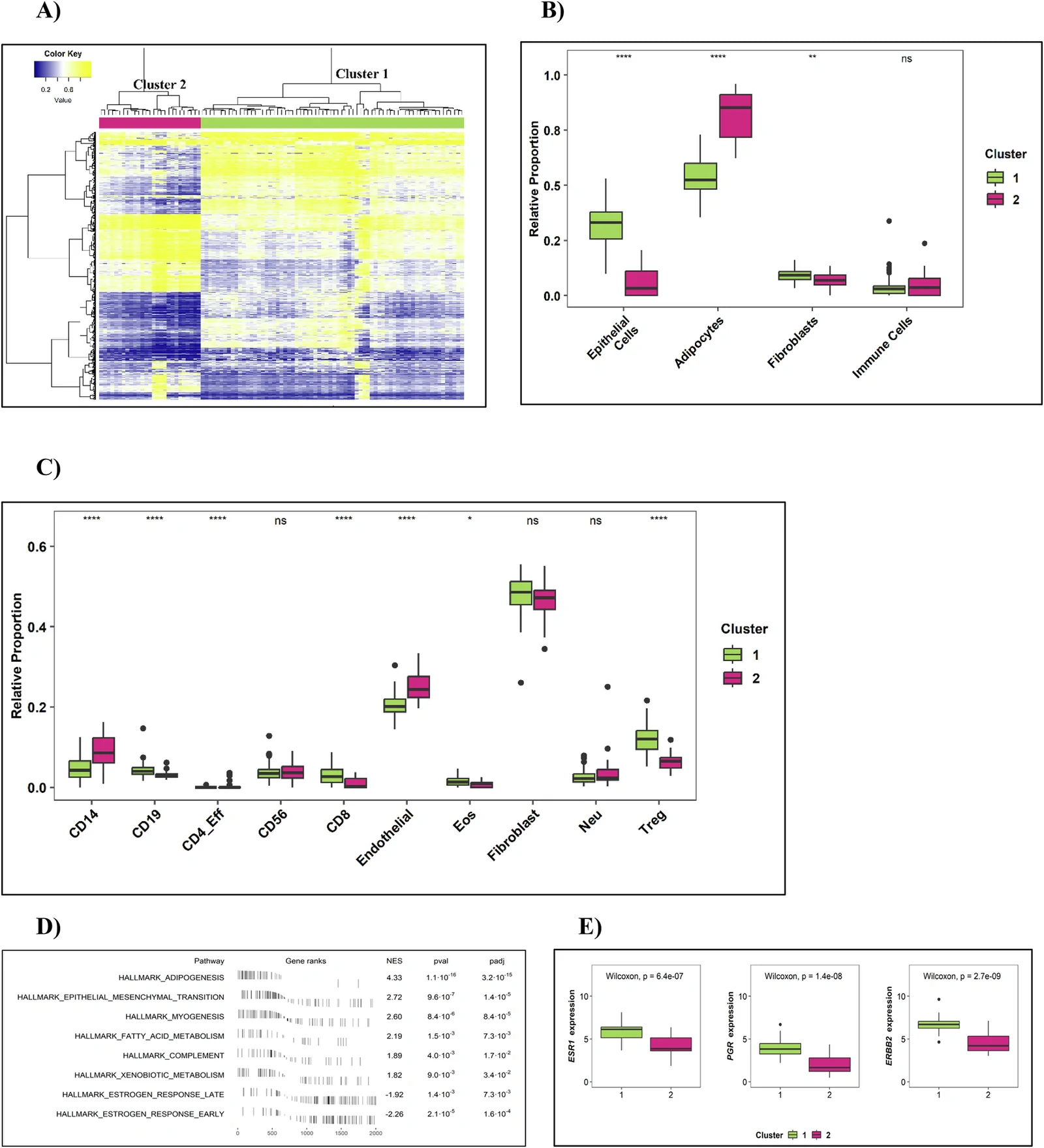
Characterization of 97 methylation profiles from adjacent normal tissue samples collected from The Cancer Genome Atlas women diagnosed with breast cancer. **A** Unsupervised clustering analysis using 10,000 most variable methylation probes; samples are represented as columns and methylation probes as rows, with beta values displayed using a color gradient from navy (*β* = 0) to yellow (*β* = 1) to indicate increasing methylation; **B** Proportions of cell types inferred by EpiDISH; **C** Proportions of cell types inferred by MethylCIBERSORT; **D** Fast Gene Set Enrichment Analysis (FGSEA) of differentially expressed genes using Hallmark gene sets from The Molecular Signatures Database (MSigDB) comparing Cluster 2 versus Cluster 1 samples; **E** Gene expression levels of *ESR1*, *PGR*, and *ERBB2* in adjacent normal tissue samples by cluster. Significance levels are denoted as follows: ***P-value < 0.001, **P-value < 0.01, *P-value < 0.05.

We conducted a parallel analysis of NAT samples from 97 breast cancer patients in TCGA and identified two distinct clusters (see Fig. 6A) that mirrored the TME-related differences observed in the HKBC cohort. Specifically, Cluster 2 (*N* = 27) was characterized by a higher content of adipocytes but significantly fewer epithelial cells, as estimated by the EpiDISH method (see Fig. 6B), compared to Cluster 1 (*N* = 70). MethylCIBERSORT data further revealed that Cluster 2 samples had elevated levels of CD14 content and endothelial cells, but reduced levels of CD8 + T and Treg cells compared to Cluster 1 samples (see Fig. 6C). FGSEA analyses based on top differentially expressed genes revealed eight significantly enriched pathways. Cluster 2 showed downregulation of early/late estrogen response pathways, alongside upregulation of adipogenesis, epithelial-mesenchymal transition, myogenesis, fatty acid metabolism, and xenobiotic metabolism pathways (see Fig. 6D, E). Correspondingly, Cluster 2 samples had much lower expression levels of the *ESR1*, *PGR*, and *ERBB2* genes compared to samples in Cluster 1 (see Fig. 6E). Finally, consistent with results based on HKBC study, TCGA NAT samples in Cluster 2 also displayed elevated levels of CD14 downstream inflammatory markers (*IL-6*, *IL-8*, and *CCL3*, Supplementary Fig. 14A), and macrophage markers (*CD68* and *CD163*, Supplementary Fig. 14B), CIBERSORTx estimates based on RNA-Seq data confirmed a more inflammatory immune landscape in Cluster 2, with higher fractions of monocytes and M2 macrophages and reduced CD8 + T cell content compared to Cluster 1 (Supplementary Fig. 14C).

## Discussion

This study aimed to characterize the molecular heterogeneity of histologically normal adjacent tissues in breast cancer patients, with a focus on epigenomic and transcriptomic alterations and their implications for tumor evolution and progression. Through unsupervised clustering of DNA methylation and gene expression profiles, we identified two distinct subtypes of NATs. Cluster 1 was enriched for epithelial and fibroblast content and exhibited higher estrogen receptor signaling, while Cluster 2 exhibited increased adiposity, immune cell infiltration, and a more chronic inflammatory tumor microenvironment. These results are consistent with our previous WGS analysis in which NAT samples in the shared-tree group, those sharing mutations with matched tumor samples, were more prevalent in Cluster 2 samples and showed greater mutational burden and exhibited a more inflammatory TME. The pro-inflammatory characteristics of Cluster 2 NAT samples are also consistent with pan-cancer study findings by Aran et al., who identified shared pro-inflammatory signals in NATs across multiple cancer types^6^, suggesting this may be a common feature of tumor-adjacent tissues. Collectively, this convergence of epigenetic and microenvironmental evidence supports the idea that molecular heterogeneity in NATs, including both genetic and epigenetic alterations, may be associated with tumor evolutionary trajectories and could potentially contribute to inter- and intra-tumoral heterogeneity.

We observed significant differences in cell composition between the two clusters. While some of this variability could stem from technical sampling differences, several lines of evidence point to underlying biological distinctions. Notably, patients with Cluster 1 NAT samples were more likely to have higher mammographic density, a systemic attribute rather than a feature confined to local tissue, indicating that cluster differences are unlikely to be driven solely by sampling variability.

Cluster 1 NAT samples had higher epithelial cell content, whereas Cluster 2 samples showed consistently higher levels of inflammation, as indicated by more severe stromal disruption, increased fat content, elevated macrophage infiltration, and increased expression of inflammatory markers, compared with the Cluster 1 samples. H&E-based tissue analysis confirmed that Cluster 2 NAT samples demonstrated significantly higher stromal disruption compared to Cluster 1 (Fig. 4D). As we have previously defined^3^, stromal disruption is a morphological metric combining reduced dense stroma-to-epithelial ratio with increased stromal cellular density, reflecting extracellular matrix alteration, chronic inflammation, and wound response processes. The elevated macrophage markers (*CD68*, *CD163*), monocytes, M2 macrophages, and inflammatory markers (*IL-6*, *IL-8*, *CCL3*) observed in our Cluster 2 NAT samples are consistent with this stromal disruption phenotype. In our prior work examining over 9000 women across the breast cancer continuum, we demonstrated that progressive stromal disruption in both normal and benign breast tissues was associated with elevated risk of subsequent aggressive (high-grade) breast cancer and was characterized by heightened innate (CD68 +), adaptive (CD3 + CD4 + , CD3 + CD8 +), immunoregulatory (CD3 + CD4 + FOXP3 +), and immune escape (PD1 + PDL1 +) pathways^3^. Notably, substantial stromal disruption was associated with poor prognosis, specifically among ER-positive patients^3^, suggesting that the molecular heterogeneity we identified in NATs may have clinical implications worth exploring in future large studies with outcome data.

Previously published studies examining NAT heterogeneity are consistent with our observations. Sun et al. investigated the relationship between TME gene expression in NAT samples and mammographic density in breast cancer cases^13^. The authors reported that their ‘inactive extratumoral subtype’ (associated with high expression of cellular adhesion genes) displayed a higher mammographic dense area, more stroma, less adipose tissue, and stronger estrogen response^13^, features that parallel our Cluster 1. Conversely, the metabolic features enriched in our Cluster 2 NAT samples, including adipogenesis and fatty acid metabolism pathways, parallel the ‘metabolic subtype’ identified by Gadaleta et al. in morphologically normal breast tissue^5^. Their metabolic subtype, characterized by lipid metabolism dysregulation and hypoxia-related events, was notably associated with poor prognosis (HR 6.1, *p* < 0.001). This parallel, along with our observation of elevated myeloid and macrophage infiltration in Cluster 2, suggests that metabolic reprogramming and immune infiltration in NAT resemble features previously linked to prognosis and potentially indicate the presence of tumor-associated macrophages.

Consistent with these microenvironmental differences, we observed differences in promoter methylation patterns in several canonical breast cancer-related genes. Notably, differential methylation of *RASSF1*, *HOXA5*, and *BRCA1* aligned with inflammatory remodeling and genomic instability signatures, supporting the presence of biologically meaningful epigenetic heterogeneity in NATs. Although the distributions of chronological age were similar between the two clusters, Cluster 2 patients exhibited a younger epigenetic age. Prior studies suggest that a younger epigenetic age is associated with a higher proportion of stem-like or progenitor cells ^14^, potentially reflecting differential cellular response to nearby tumor cells. Additionally, differences in the immune cell composition might underline this discrepancy, consistent with findings from studies associating immune cell types with epigenetic age acceleration in blood^15^. In contrast, Cluster 1 samples with higher epithelial content may represent a more pre-neoplastic state, potentially exhibiting accelerated epigenetic aging as an early transformation event. This is in line with our earlier work associating high estrogen receptor (ER) levels with accelerated methylation aging^13^.

Our current findings further support a link between the TME and tumor evolutionary trajectories^10^. Although this analysis did not directly assess evolutionary history due to the absence of matched genomic data in all cases, the patterns observed mirror those from our earlier WGS-based phylogenetic analysis. Specifically, patients in Cluster 2 were more likely to have shared mutations between paired tumor and NAT samples, while patients in Cluster 1 were more likely to exhibit distinct evolutionary trajectories. Our previous analysis revealed that NAT samples in the shared-tree group had a higher number of mutations and a more inflammatory TME, consistent with the current study’s findings. Together, the associations between NAT inflammation, shared mutations, and tumor evolutionary features underscore the potential of NAT profiling in informing early events in carcinogenesis.

Our study has several limitations. First, although our cohort is larger than most previous omics-based NAT studies, the sample size, particularly for RNA-seq data, remains modest. Second, the study population was limited to Chinese patients from two hospitals, which may limit the generalizability of the findings in the broader Chinese population. Third, the small sample size also hindered our ability to assess associations with clinical outcomes, such as overall survival in both HKBC and TCGA cohorts. Fourth, while our NAT classification was independent of PAM50 tumor subtypes, supporting its distinct biological basis, this lack of association may limit immediate clinical translatability, as current treatment algorithms are largely guided by intrinsic subtype classification. Future studies with larger cohorts will be needed to determine whether NAT classes provide complementary prognostic or predictive value alongside established molecular subtypes. Importantly, our findings are observational and do not establish whether NAT phenotypes drive, result from, or co-evolve with tumor development. Finally, only a subset of patients had WGS data; therefore, any findings related to tumor evolution should be interpreted with caution. Despite these limitations, we successfully replicated our primary findings in the TCGA dataset derived from a different population with distinct genetic and environmental backgrounds, enhancing confidence in the robustness of our results.

In conclusion, our study provides additional insights into the molecular heterogeneity of NATs in breast cancer patients. We identified two distinct subtypes, each associated with unique epigenomic and transcriptomic alterations. The more inflammatory phenotype seen in Cluster 2 may be associated with tumor evolution and warrants further investigation as a potential indicator of tumor-microenvironment interaction during carcinogenesis. These findings extend the concept of field cancerization and highlight the importance of including NATs in studies of tumor evolution. Future research should prioritize longitudinal studies to monitor molecular alterations in NATs over time and incorporate spatial profiling and multi-omics integration, such as proteomics and metabolomics, to better characterize the complex interplay between the TME and cancer development.

## Methods

### Hong Kong breast cancer (HKBC) study participants

The study design and biospecimen collection have been previously described in detail^16^. Briefly, fresh frozen breast tumor samples and corresponding NATs were obtained from treatment-naïve breast cancer patients who were diagnosed and treated at two hospitals in Hong Kong between 2013 and 2016. Inclusion criteria for participants were: (a) female sex, (b) age between 20 and 84 years, (c) a breast cancer diagnosis confirmed histologically within three months before the recruitment interview (International Classification of Diseases, Tenth Revision, code 50), and (d) Chinese ethnicity with a minimum of 5 years of residency in Hong Kong. Patients who had received pre-surgical treatment were excluded from the study. Clinical characteristics and breast cancer risk factors were gathered through medical records and questionnaires. Mammographic density phenotypes such as dense area, non-dense area, and percent density were estimated in a subset of women (*N* = 70) using the Laboratory for Individualized Breast Radiodensity Assessment (LIBRA) software, version 1.0.4. The current analysis involved 188 patients with methylation profiling data available for paired tumor and NAT samples. The research protocol received approval from the ethics committees of the Joint Chinese University of Hong Kong-New Territories East Cluster, the Kowloon West Cluster (IRB approval #2014.238), and the National Cancer Institute (NCI), National Institutes of Health (NIH) (IRB approval #13CN137), and was conducted in accordance with the Declaration of Helsinki. All participants provided written informed consent prior to surgery.

To replicate our findings based on HKBC, we also extracted DNA methylation 450K array (*N* = 97) and RNA gene expression data (*N* = 111) from breast NAT samples from The Cancer Genome Atlas (TCGA).

### Methylation profiling and cell deconvolution

Methylation profiling was conducted on 188 NAT samples and 196 tumor samples (188 pairs) using the Infinium MethylationEPIC BeadChip (EPIC array, also known as the 850 K array; Illumina, San Diego, CA) at the Cancer Genomics Research Laboratory (CGR), NCI, NIH. Among them, RNA-seq data were available for 177 tumor samples, and 76 NAT samples (71 pairs) and paired germline/NAT/tumor whole-genome sequencing (WGS) data were available for 41 patients (Supplementary Fig. 1). Data preprocessing and normalization were performed using the R package minfi^17^. Raw methylated and unmethylated intensities were background-corrected and dye-bias-equalized to address technical variations in signal across arrays. Probes were excluded from further analyses for the following reasons: (a) probes mapping to sex chromosomes (*N* = 19,627), (b) probes where the targeted CpG site coincides with a common single-nucleotide polymorphism (*N* = 28,838), and (c) probes that do not map uniquely to the human reference genome hg38 (*N* = 41,850). A total of 776,521 probes were retained for subsequent analysis. We identified the top 10,000 most variable sites by ranking all methylation probes by β value variance across all samples. The Horvath, Hannum, and Levine clocks were employed to estimate epigenetic ages and age accelerations as previously described^18–20^. DNAm age acceleration was estimated to be the residual from regressing DNA methylation age on chronological age. Additionally, EpiDISH^21^ and MethylCIBERSORT^22^ were utilized to estimate cellular composition based on the methylation data. While MethylCIBERSORT primarily focuses on stromal and immune cell subpopulations, EpiDISH provides estimates for a broader range of cell types within a bulk tissue sample, encompassing both immune and non-immune cells. The breast tissue-specific methylation signature matrices available in these packages were used. For MethylCIBERSORT, we used an additional step of the CIBERSORTx (https://cibersortx.stanford.edu/) in relative mode on our DNA methylation data with 1000 permutations and without quantile normalization.

TCGA methylation data from paired tumor and normal samples, profiled using Illumina HumanMethylation450 BeadChip array, were subjected to identical data quality control procedures as those taken in the HKBC study.

### Gene expression-based analyses

Among 188 patients with paired tumor/NAT methylation data, RNA-seq data (Illumina HiSeq 4000) were available from 76 NAT and 177 tumor samples (Supplementary Fig. 1)^10,23^. Gene expression was quantified in transcripts per million (TPM), with log2TPM utilized for statistical analyses. CIBERSORTx^24^ was employed to estimate the relative proportions of 22 immune cell types from the gene expression profiling data. PAM50 subtypes—luminal A, luminal B, HER2-enriched, basal-like, and normal-like—were determined using the Absolute Intrinsic Molecular Subtyping (AIMS) method^11^ based on the RNA-seq data.

### Tissue morphology based on H&E-stained sections

We obtained archived hematoxylin and eosin (H&E)-stained sections from a subset of the patients (*N* = 115, Supplementary Fig. 1) included in the current methylation analysis. Digitized images of these sections were stored and analyzed at the Molecular and Digital Pathology Laboratory at CGR, NCI, NIH. Supervised machine learning algorithms based on the Halo© (Indica Labs, Albuquerque, NM) random forest algorithms were applied to these H&E-stained sections to perform tissue (epithelial, stromal, adipose), stromal (dense collagenous versus loose and/or remodeled), and cell (immune and non-immune) classification tasks^3,25^. As previously described^3^, data from tissue/stromal classification (i.e., dense stroma-to-epithelium ratio) and cell detection (i.e., stromal cellular density) algorithms were used to define stromal disruption as follows: 1) none/minimal stromal disruption (high dense stromal-to-epithelial ratio and low stromal cellular density; high dense stromal-to-epithelial ratio and intermediate or high stromal cellular density; intermediate dense stromal-to-epithelial ratio and low or intermediate stromal cellular density; low dense stromal-to-epithelial ratio and low stromal cellular density); 2) moderate stromal disruption (intermediate dense stromal-to-epithelial ratio and high stromal cellular density, or vice versa); and 3) substantial stromal disruption (low dense stromal-to-epithelial ratio and high stromal cellular density)^3^.

### Statistical analysis

We performed unsupervised clustering analysis on DNA methylation data using the 10,000 most variable probes to identify distinct sample groupings of 188 NATs. For the hierarchical clustering, Euclidean distance was calculated, and Ward’s minimum variance method with squared distances (ward.D^2^) was applied to group samples and probes into compact clusters. Uniform manifold approximation and projection (UMAP) was then utilized for validating and visualizing the clusters^26^. The parameters for UMAP were set as follows: n_neighbors = 30 and min_dist = 0.01, which were chosen based on preliminary tests to optimize cluster separation. To assess the optimal number of clusters, we performed consensus clustering analysis. The consensus matrix heatmap at *k* = 2 showed well-defined clusters with high intra-cluster consensus, whereas the *k* = 3 solution captured additional, albeit more subtle, heterogeneity (Supplementary Fig. 2). Overall, the consensus clustering results support either *k* = 2 or *k* = 3, with a modest preference for *k* = 2 based on cluster stability and interpretability. Accordingly, we focused on the two-cluster solution in the primary analyses. The Wilcoxon rank-sum test was used to evaluate differences in the medians of each studied feature across the identified clusters. Fisher’s exact test was performed to investigate differences in features of a categorical nature. Univariable logistic regression was employed to identify significantly differentially expressed genes among the top 2000 variable genes between the two methylation clusters. Fast gene set enrichment analysis (FGSEA) was utilized using the R package “fgsea” along with 50 Hallmark gene sets, incorporating ranked z-scores derived from differential expression analysis of all 2,000 genes examined in our study. All statistical tests in the present study were two-sided and performed using SAS v.9.4 (SAS Institute, Cary, NC, USA) or R v.4.3.1 (R Foundation for Statistical Computing, Vienna, Austria).

## Supplementary information


Supplementary Material
Supplementary Table1


## Data Availability

The accession number for the sequencing and array data reported in this paper is the dbGaP database: phs001870.v2.p1, which can be accessed via this web link: https://www.ncbi.nlm.nih.gov/projects/gap/cgi-bin/study.cgi?study_id=phs001870.v2.p1.
