# Supplementary material for: Methylation profiling of normal tissue adjacent to breast tumors reveals two distinct groups with divergent tumor microenvironment features
Source: NPJ Breast Cancer. 2026 Jun 2;12:116. doi: 10.1038/s41523-026-00944-x (PMC13534499; doi:10.1038/s41523-026-00944-x)
Supplement: Supplementary file 1 [file 41523_2026_944_MOESM1_ESM.pdf]

Supplementary figure 1. Overview of multi-omics data availability in the Hong Kong breast cancer study.

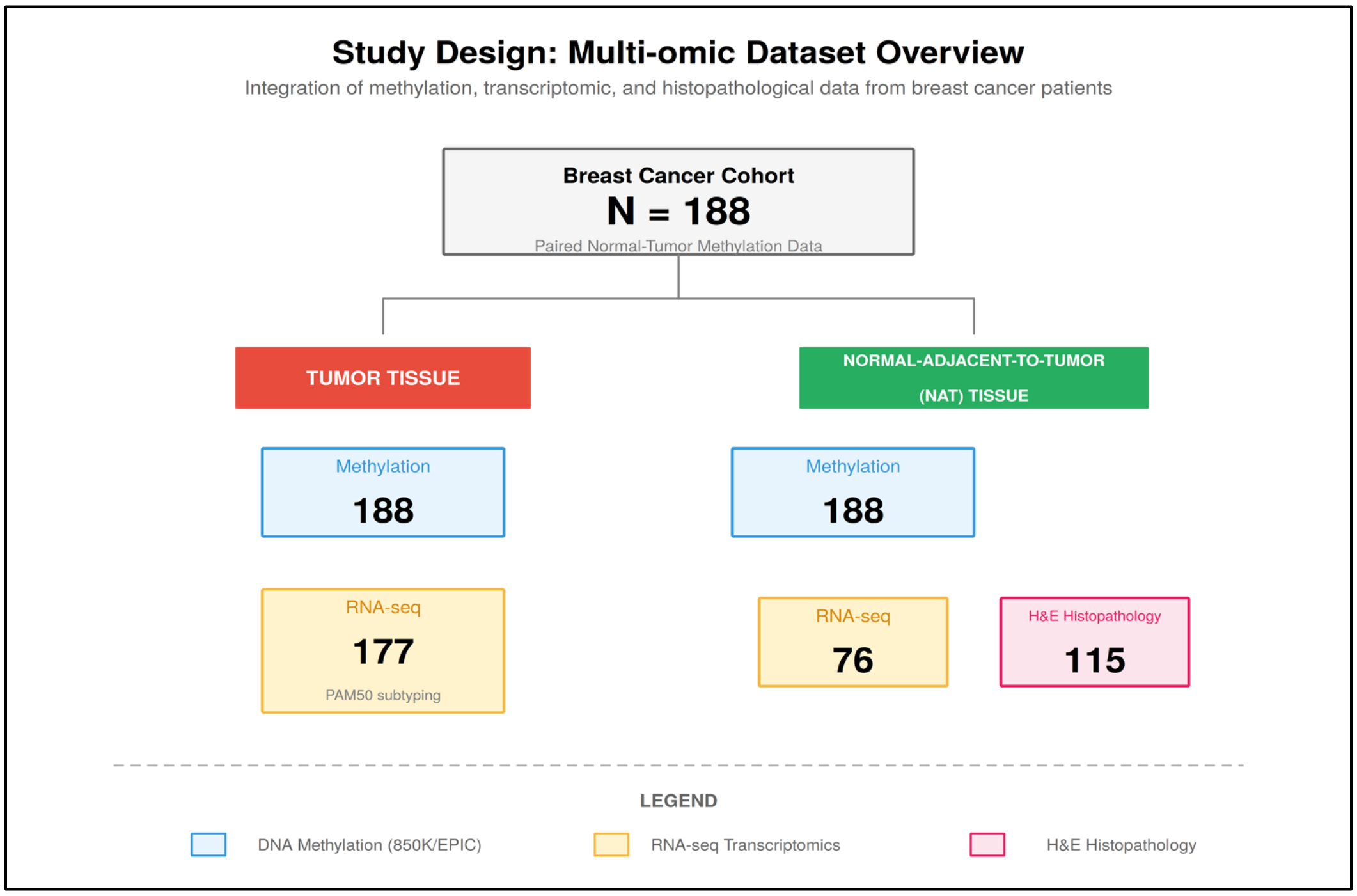

Supplementary figure 2. Consensus clustering evaluation for optimal cluster number selection.

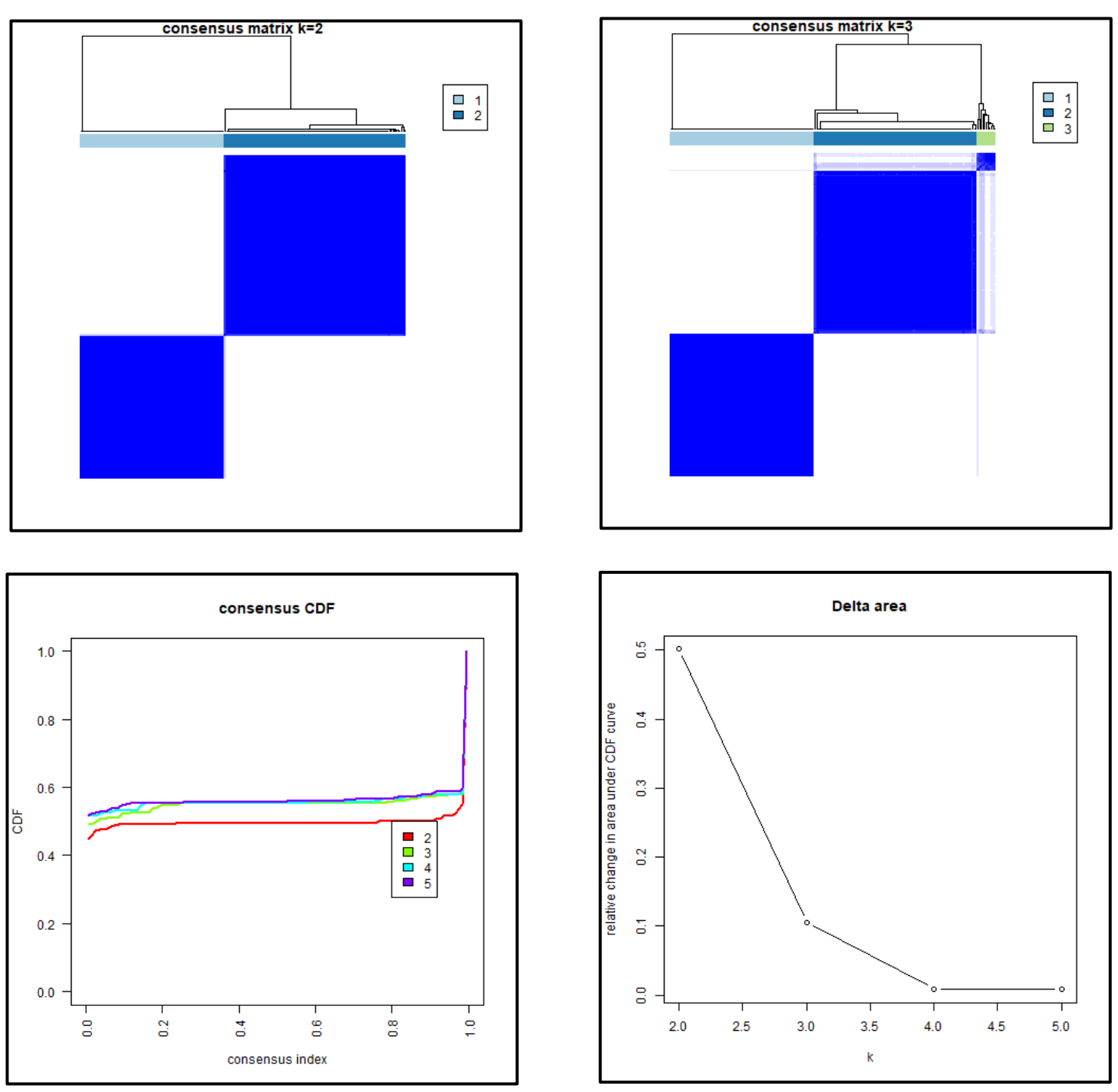

Supplementary figure 3. Stacked bar plot showing the relationship between the two methylation clusters and distances between normal and matched tumor samples. The distances between tumor and adjacent normal samples were grouped into three categories: <2, 2-4, and ≥4 cm. Green is used to annotate samples from Cluster 1 and red is used to denote samples from Cluster 2.

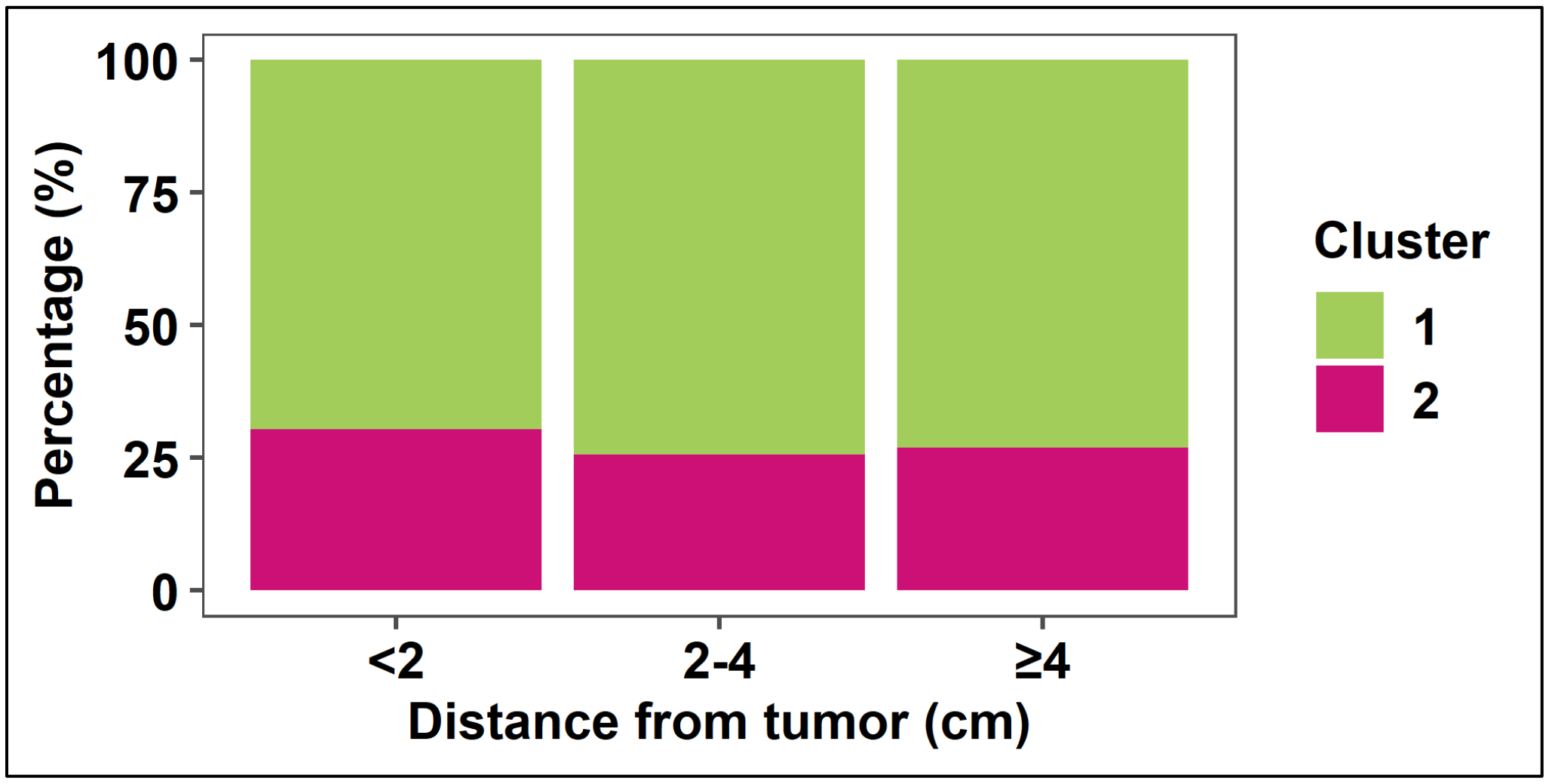

Supplementary figure 4. UMAP plot of clustering of 92 transcriptomic profiles from adjacent normal tissue samples in the Hong Kong breast cancer study using 2,000 most variable genes.

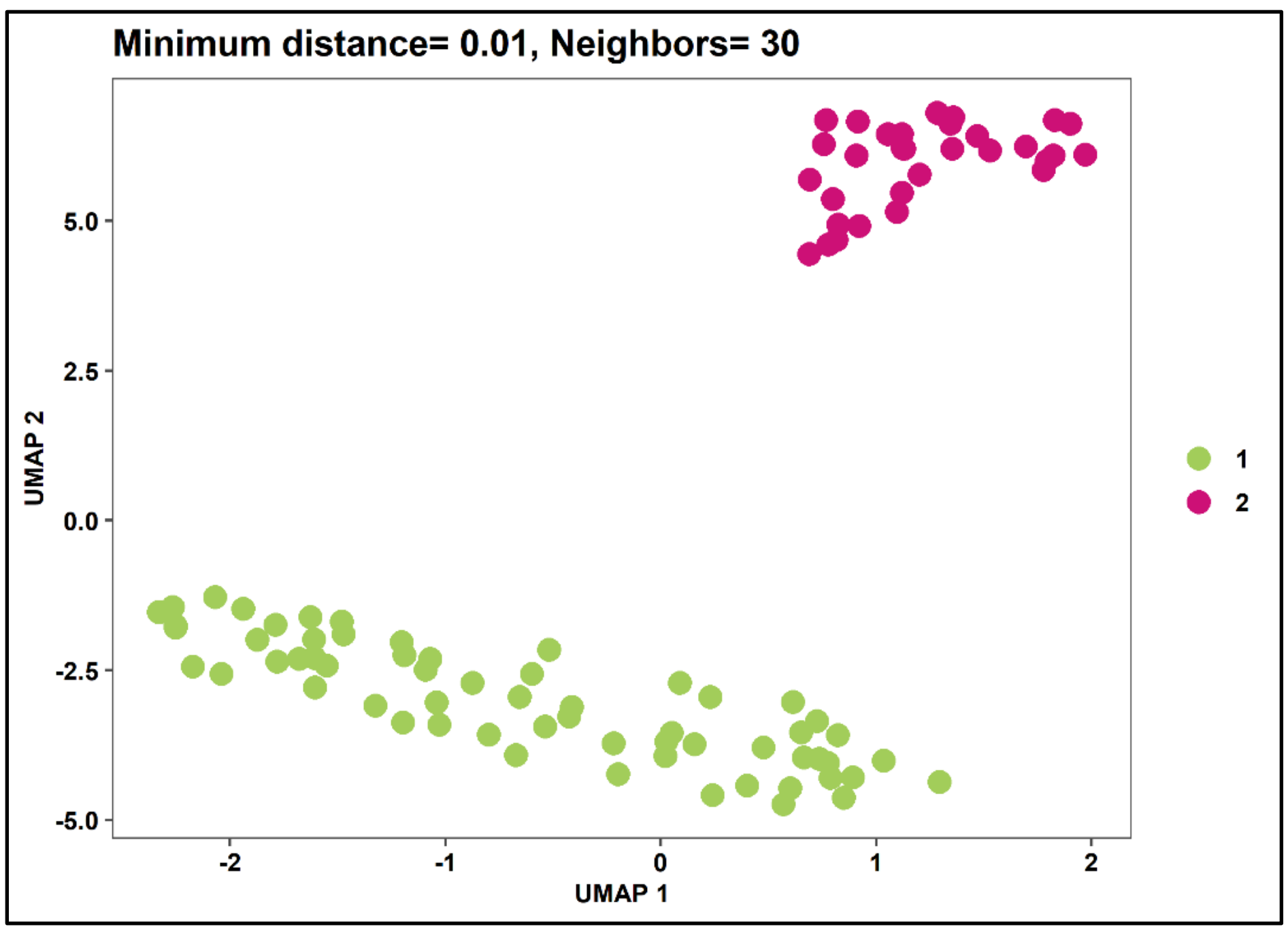

Supplementary figure 5. DNA methylation age acceleration across three epigenetic clocks by methylation cluster.

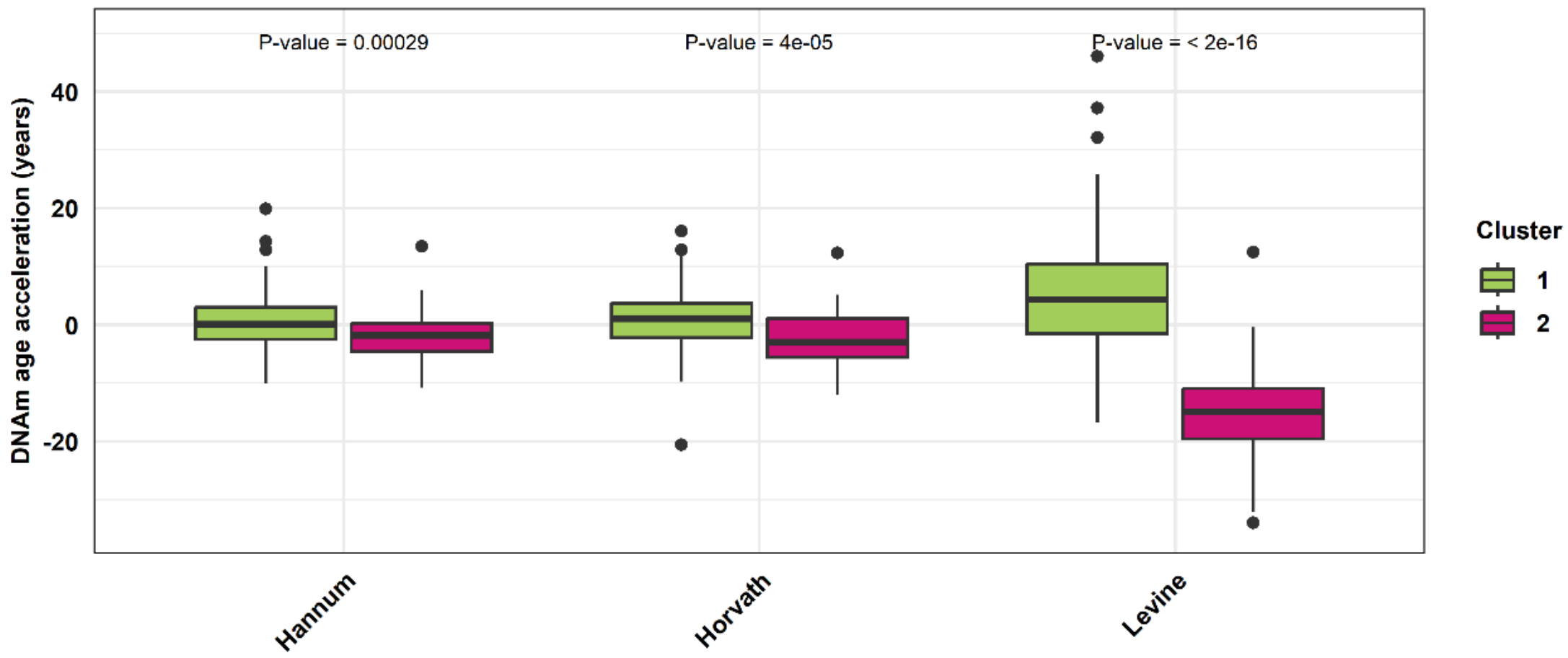

Supplementary figure 6. Expression levels of *CDKN2A* were estimated in 76 adjacent normal tissue samples in the Hong Kong breast cancer study stratified by methylation cluster.

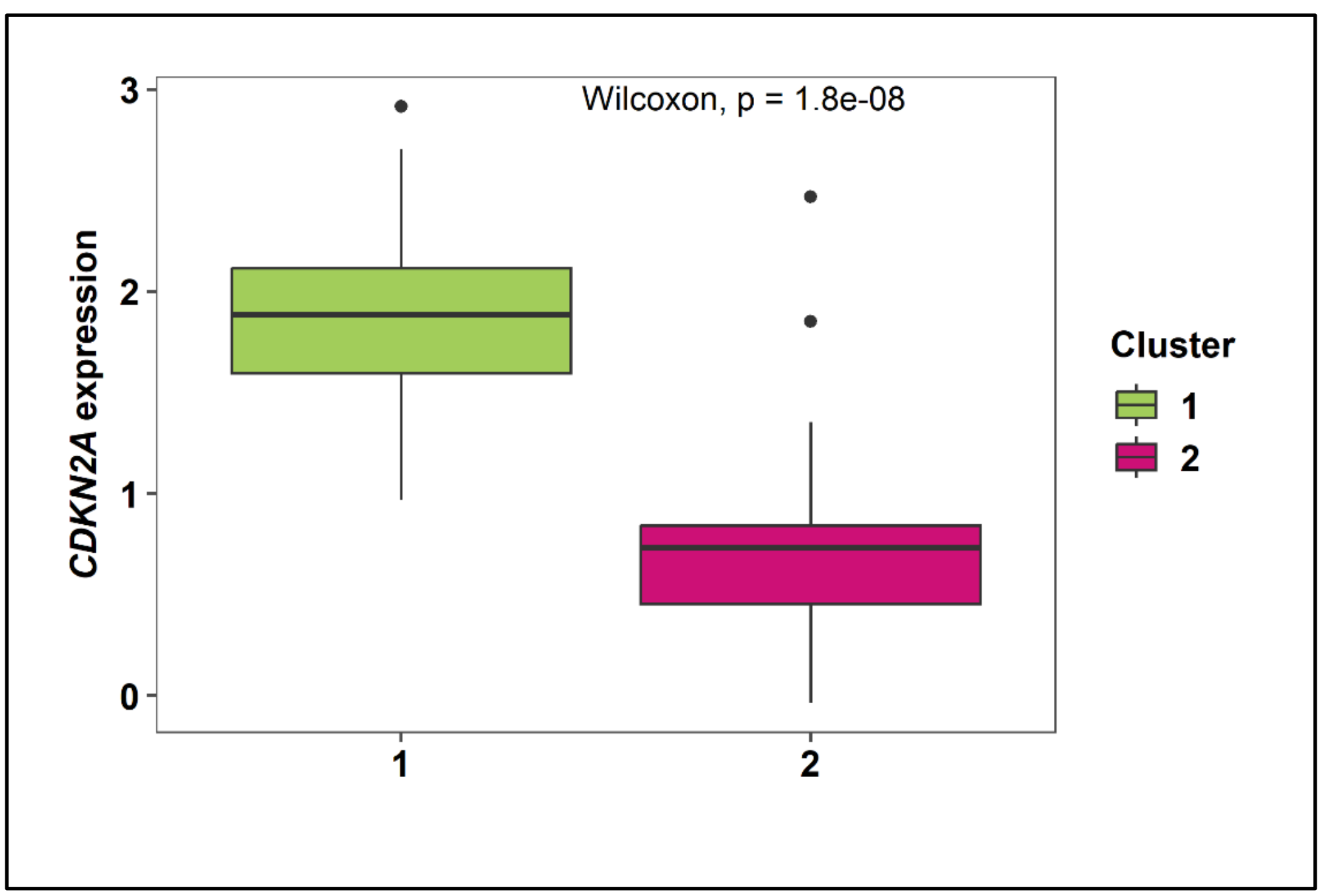

Supplementary figure 7. Representative histological images of adjacent normal tissue samples from A) Cluster 1 and B) Cluster 2.

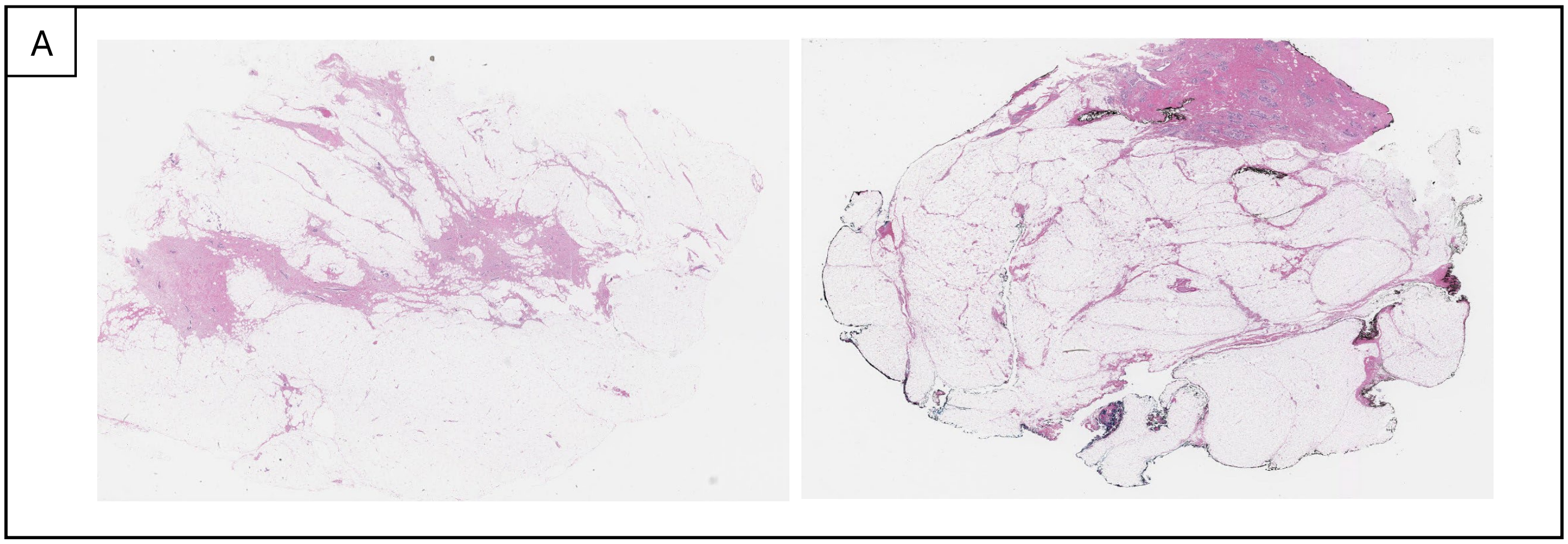

Supplementary figure 8. Expression levels of *IL-6, IL-8, CCL3* were estimated in 76 adjacent normal tissue samples stratified by methylation cluster.

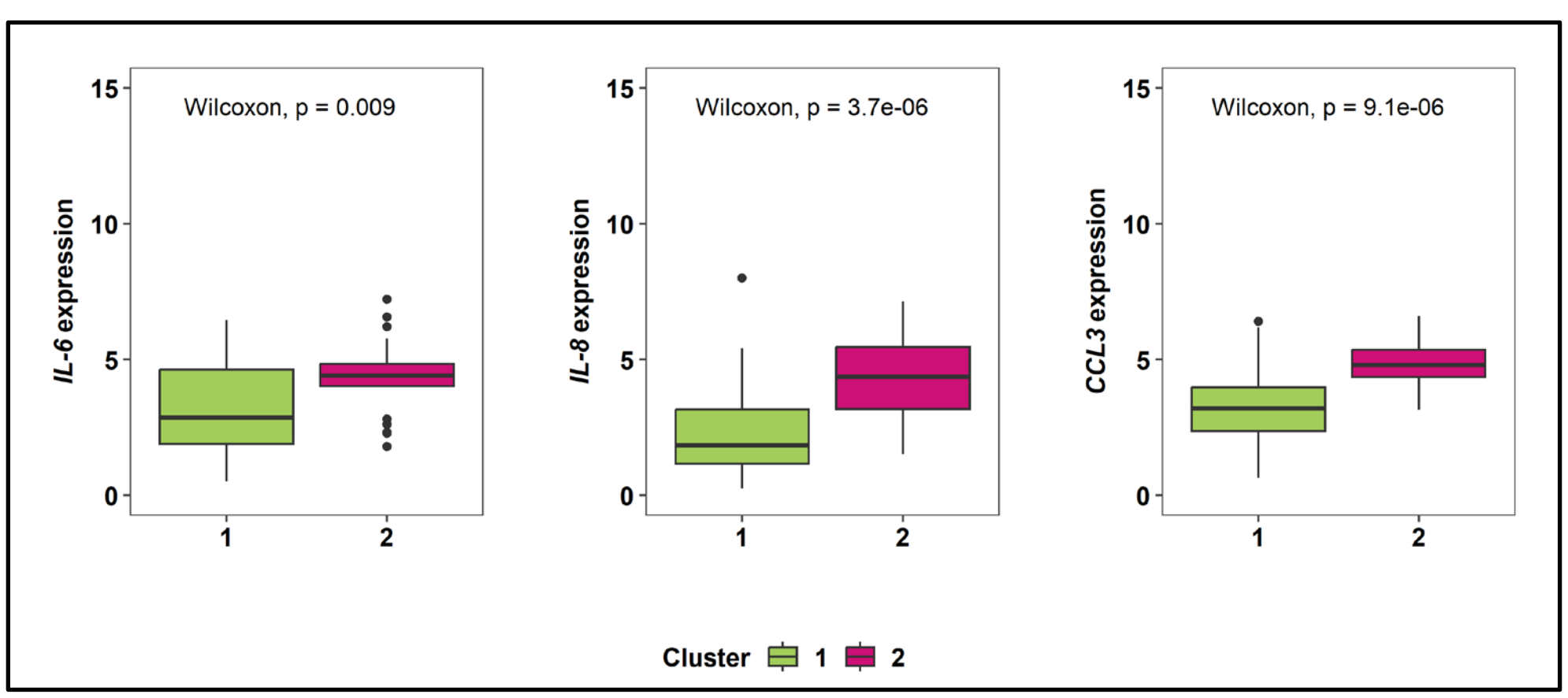

Supplementary figure 9. Proportions of immune cell subpopulations were estimated using CIBERSORTx in 76 adjacent normal tissue samples stratified by methylation cluster.

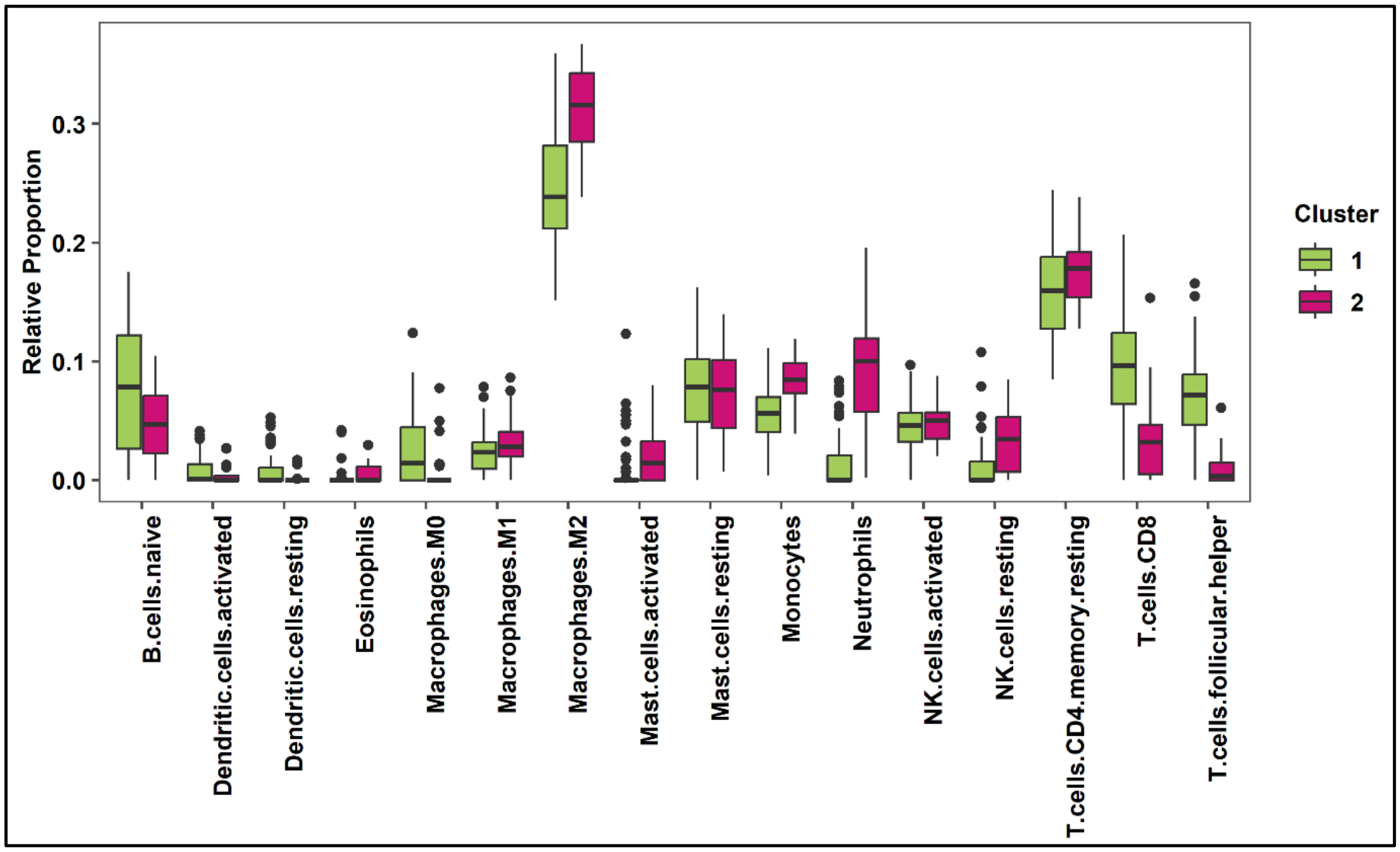

Supplementary figure 10. Expression levels of macrophage markers *CD68* and *CD163* were estimated in 76 adjacent normal tissue samples and stratified by cluster.

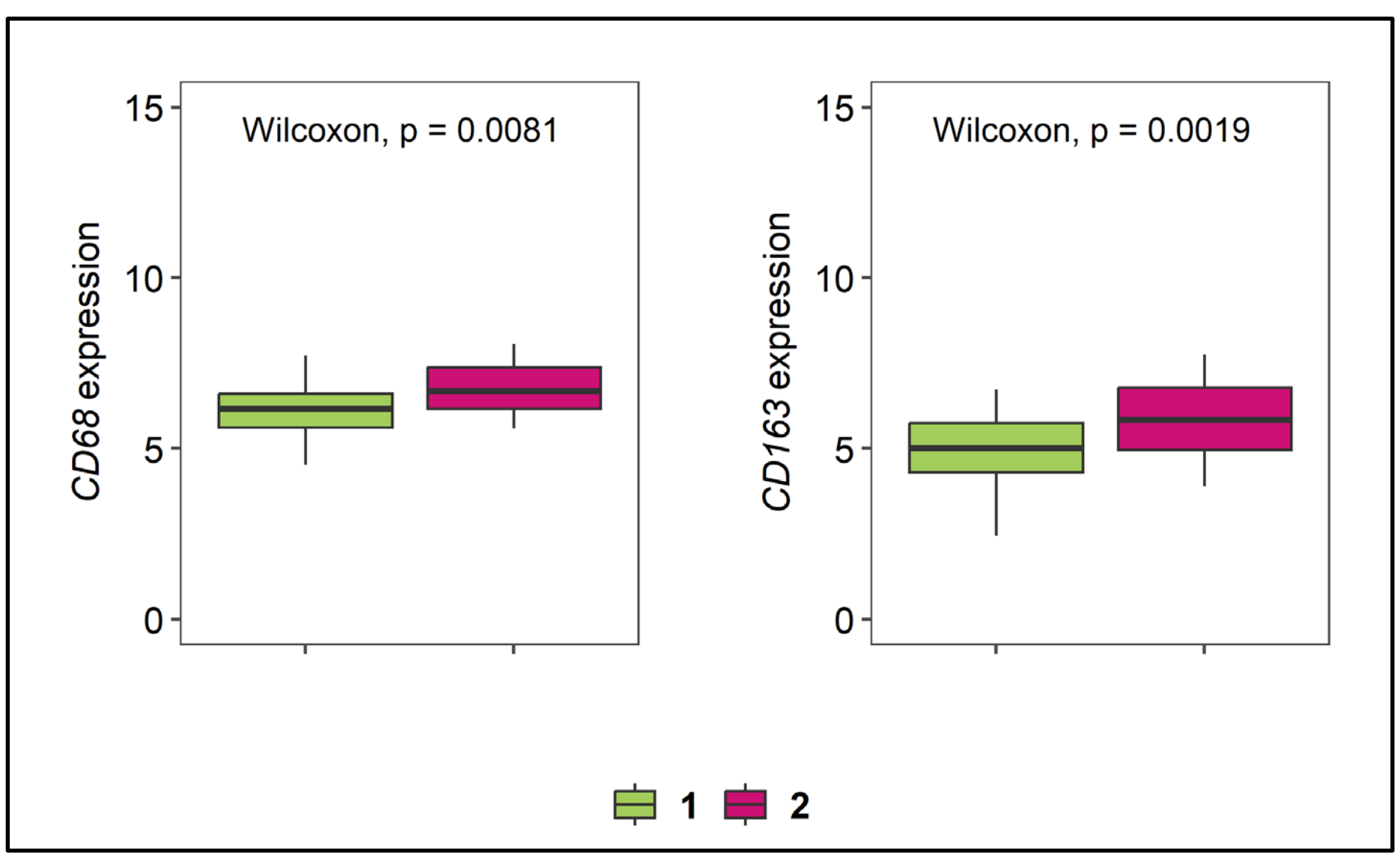

Supplementary figure 11. Stacked bar plot of methylation clusters for each phylogenetic tree group. ‘Tumor only’ group included subjects with tree structures observed in tumor tissue only, ‘Shared tree’ subjects had mutations shared between paired tumor and NAT samples, and ‘Multiple trees’ group includes subjects whose clonal/subclonal expansions in tumor were independent of those observed in normal adjacent tissue, i.e. distinct tree structures in tumor and NAT samples were observed (1). Green is used to annotate samples from Cluster 1 and red is used to denote samples from Cluster 2. The area of each rectangle is proportional to the frequency of samples in each combination of categories, for example, adjacent normal samples in the ‘Multiple trees’ group represent the largest category.

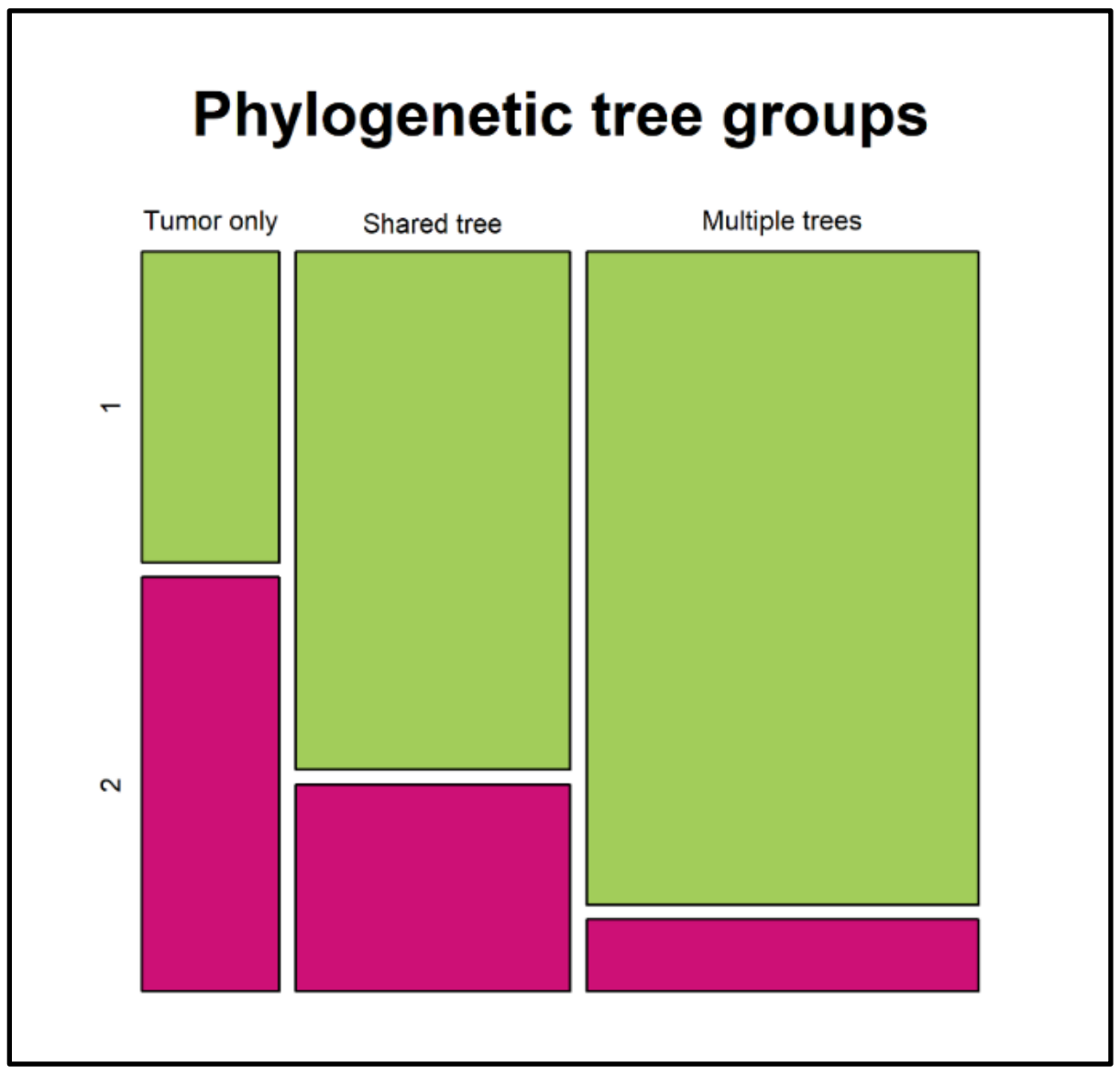

Supplementary figure 12. Promoter methylation levels of *RASSF1*, *HOXA5*, and *BRCA1* by methylation cluster.

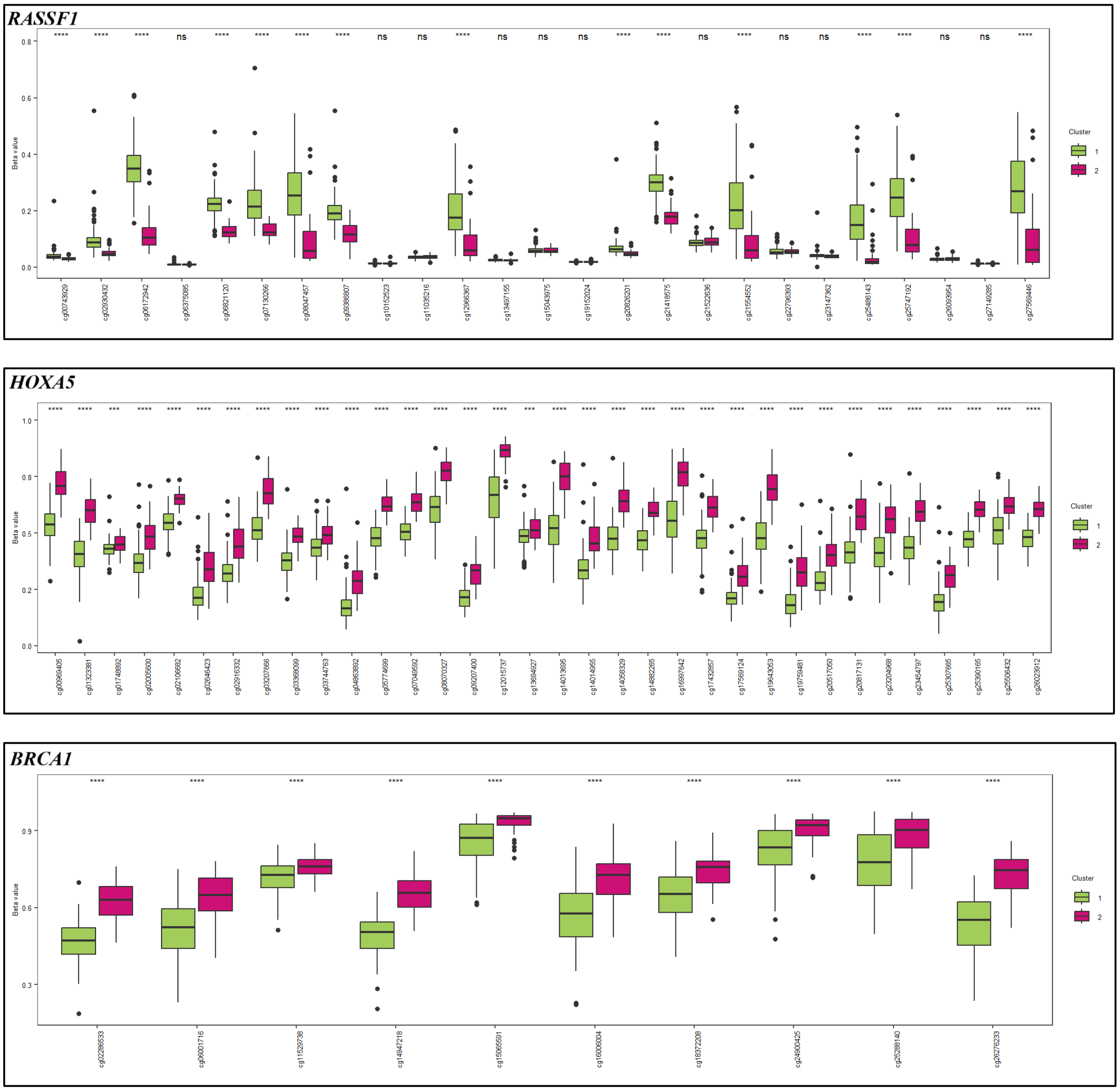

Supplementary figure 13. Three-cluster (k = 3) consensus clustering solution reveals additional heterogeneity within the epithelial-enriched NAT phenotype.

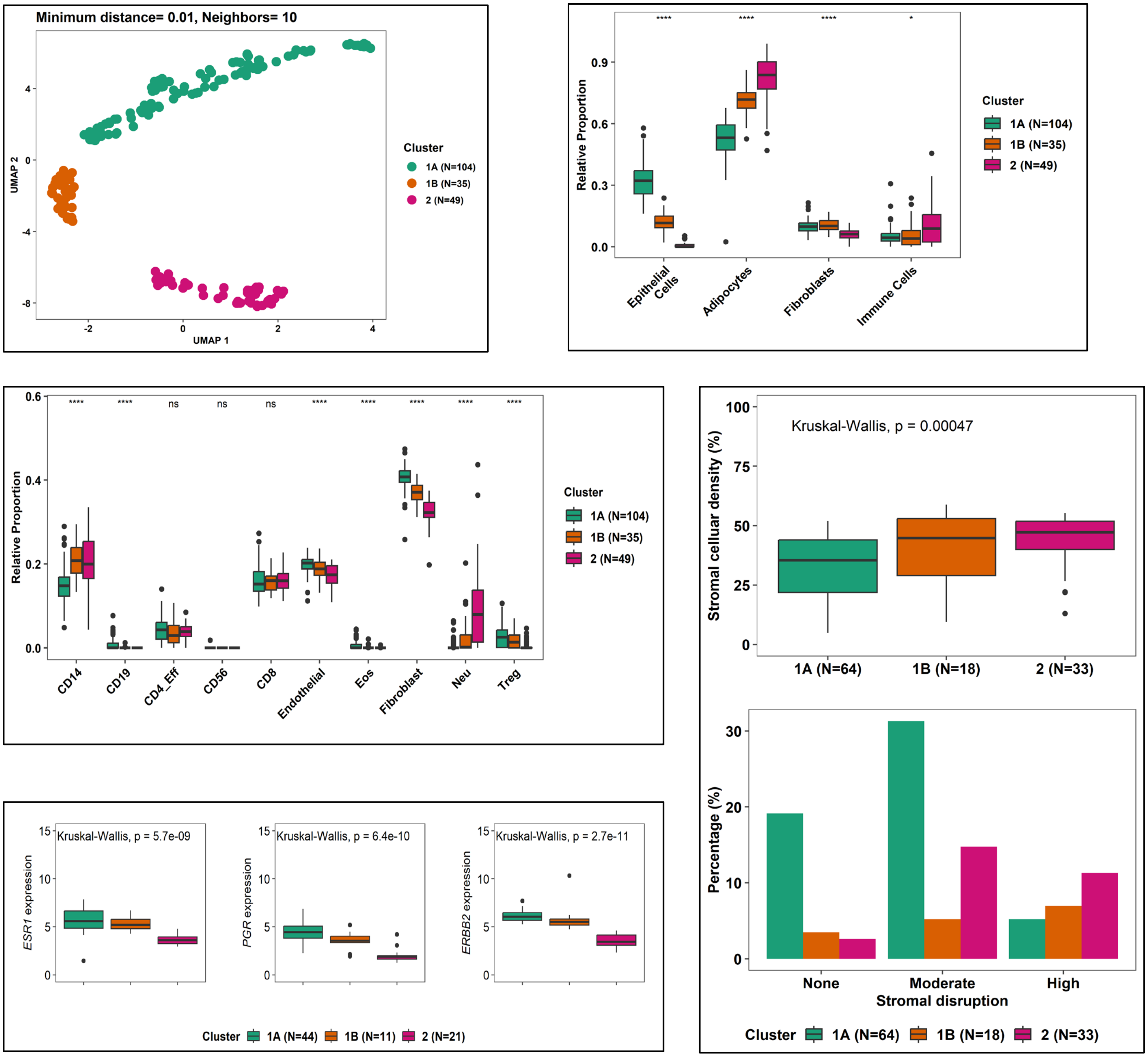

Supplementary figure 14. Expression levels of A) inflammatory markers such as *IL-6*, *IL-8*, and *CCL3*, B) macrophage markers *CD68* and *CD163* and C) relative proportions of CIBERSORTx cell populations estimated in 83 The Cancer Genome Atlas (TCGA) adjacent normal tissue samples stratified by cluster.

A)

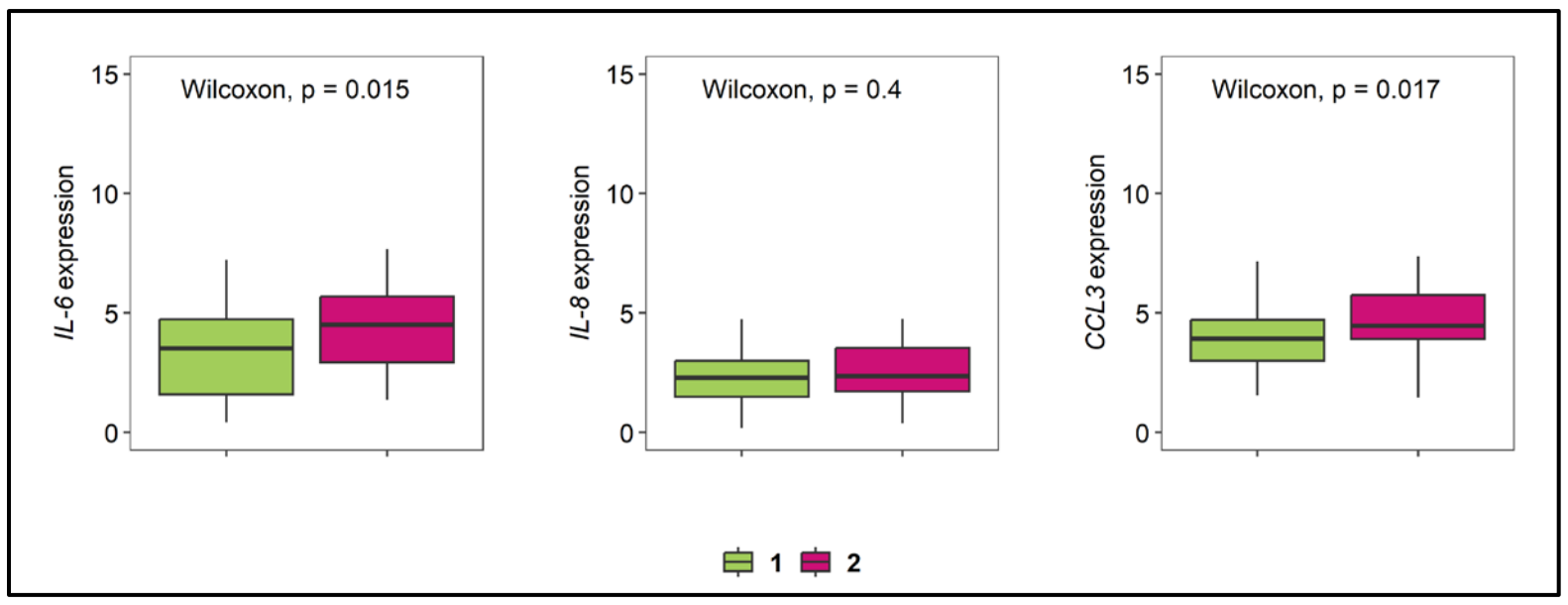

B)

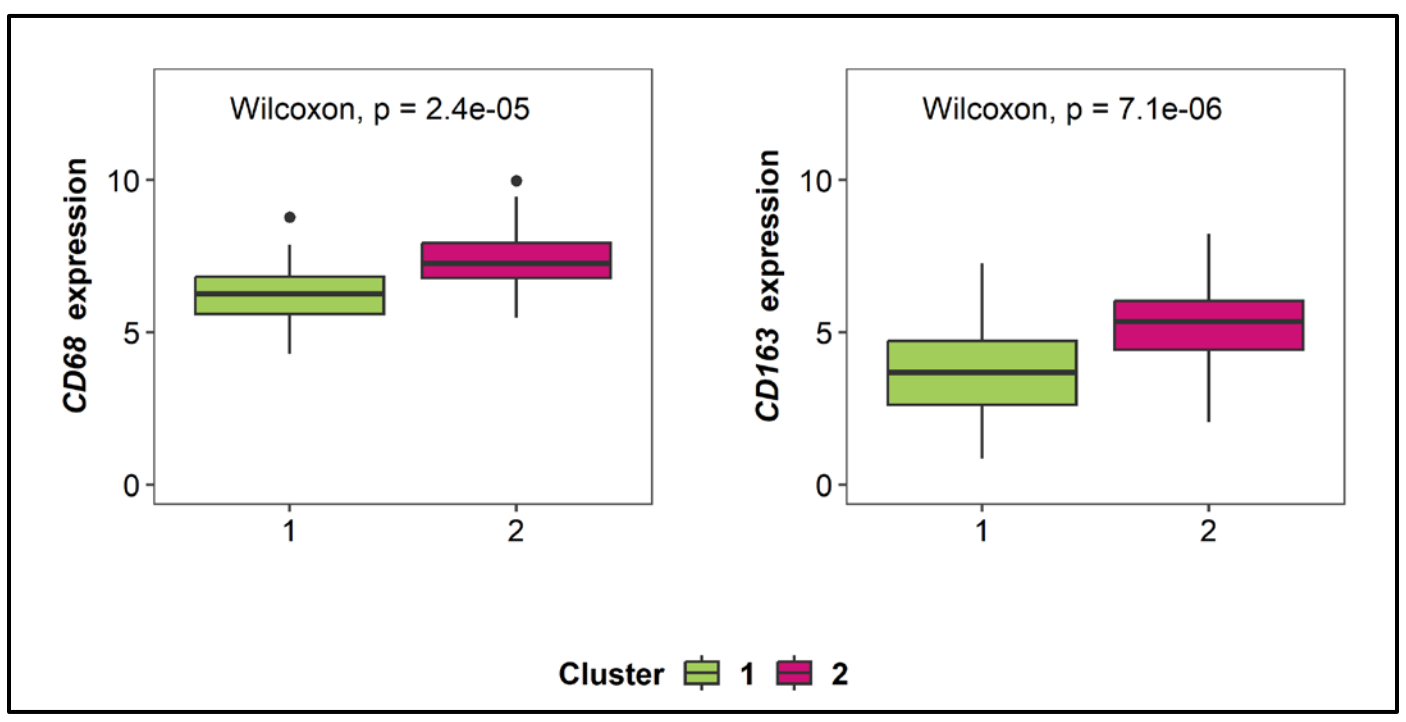

C)

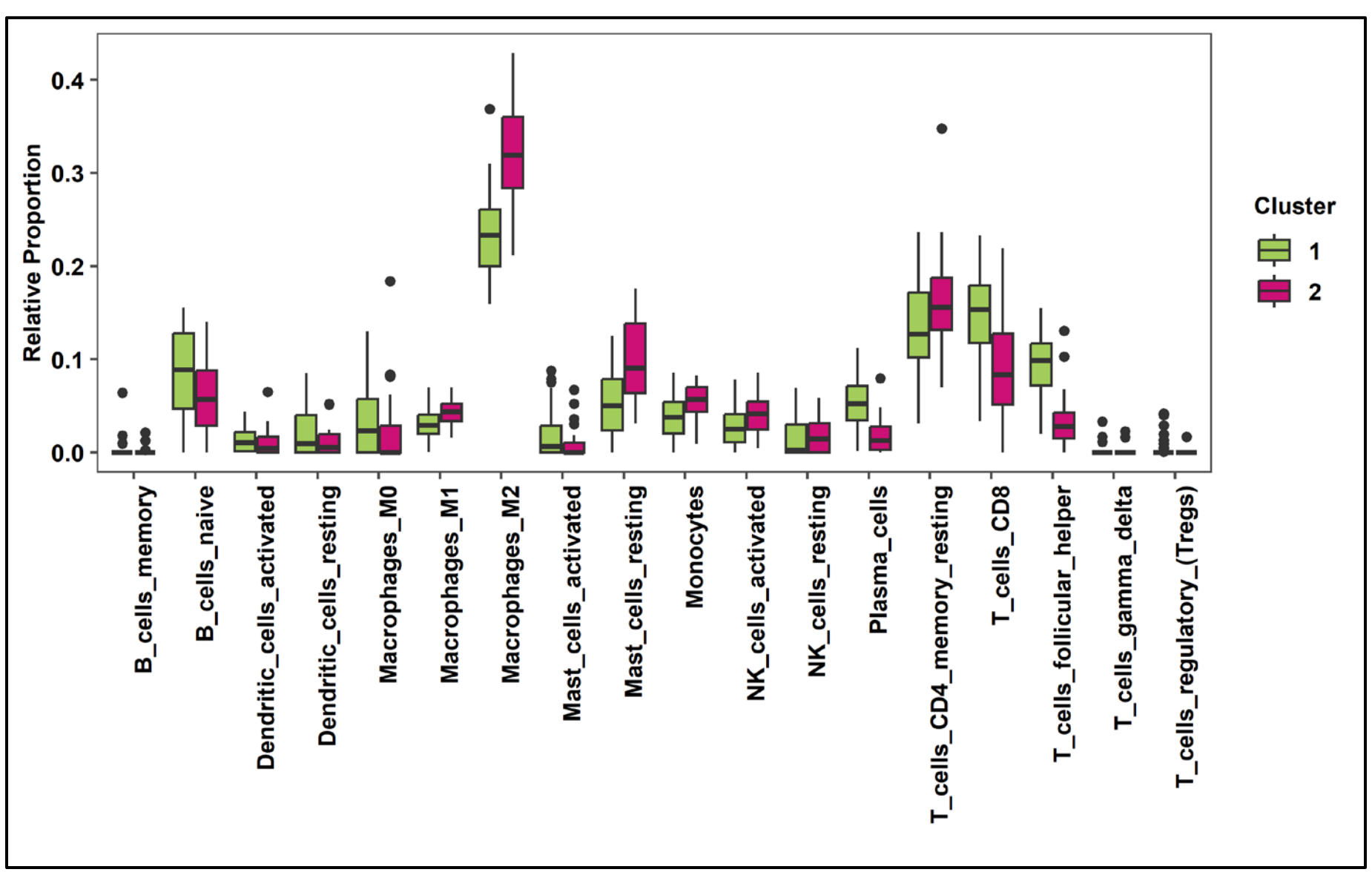

1 Zhu B, Tapinos A, Koka H, Yi Lee PM, Zhang T, Zhu W, et al. Genomes and epigenomes of matched normal and tumor breast tissue reveal diverse evolutionary trajectories and tumor-host interactions. Am J Hum Genet. 2024;111(12):2773-88.
